# Toward memristive in-memory computing: principles and applications

**DOI:** 10.1007/s12200-022-00025-4

**Published:** 2022-05-12

**Authors:** Han Bao, Houji Zhou, Jiancong Li, Huaizhi Pei, Jing Tian, Ling Yang, Shengguang Ren, Shaoqin Tong, Yi Li, Yuhui He, Jia Chen, Yimao Cai, Huaqiang Wu, Qi Liu, Qing Wan, Xiangshui Miao

**Affiliations:** 1 School of Integrated Circuits, School of Optical and Electronic Information, Wuhan National Laboratory for Optoelectronics, Optics Valley Laboratory, Huazhong University of Science and Technology, Wuhan, 430074 China; 2 Hubei Yangtze Memory Laboratories, Wuhan, 430205 China; 3 AI Chip Center for Emerging Smart Systems, InnoHK Centers, Hong Kong Science Park, Hong Kong, China; 4 School of Integrated Circuits, Peking University, Beijing, 100871 China; 5 School of Integrated Circuits, Beijing National Research Center for Information Science and Technology (BNRist), Tsinghua University, Beijing, 100084 China; 6 Frontier Institute of Chip and System, Fudan University, Shanghai, 200433 China; 7 School of Electronic Science and Engineering, and Collaborative Innovation Centre of Advanced Microstructures, Nanjing University, Nanjing, 210093 China

**Keywords:** Memristor, In-memory computing, Matrix–vector multiplication, Machine learning, Scientific computing, Digital image processing

## Abstract

With the rapid growth of computer science and big data, the traditional von Neumann architecture suffers the aggravating data communication costs due to the separated structure of the processing units and memories. Memristive in-memory computing paradigm is considered as a prominent candidate to address these issues, and plentiful applications have been demonstrated and verified. These applications can be broadly categorized into two major types: soft computing that can tolerant uncertain and imprecise results, and hard computing that emphasizes explicit and precise numerical results for each task, leading to different requirements on the computational accuracies and the corresponding hardware solutions. In this review, we conduct a thorough survey of the recent advances of memristive in-memory computing applications, both on the soft computing type that focuses on artificial neural networks and other machine learning algorithms, and the hard computing type that includes scientific computing and digital image processing. At the end of the review, we discuss the remaining challenges and future opportunities of memristive in-memory computing in the incoming Artificial Intelligence of Things era.

**Graphical Abstract:**

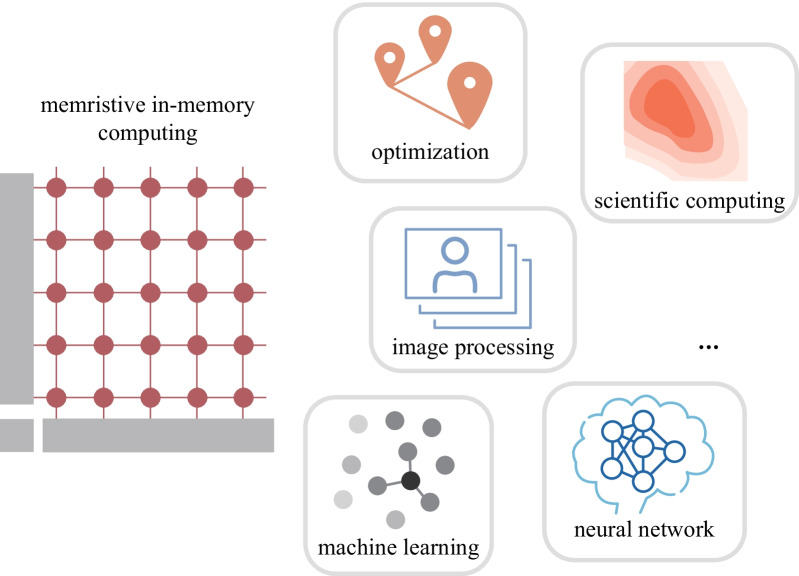

## Introduction

Over the past few decades, the development of computer science has provided the fundamentals of artificial intelligence where machine learning (ML) stands out and attracts much attention [1]. ML algorithms, including artificial neural networks (ANNs), data clustering, regression, etc., rely heavily on the data processing capability of computer systems. The von Neumann architecture demonstrates the outstanding performance to handle general data processing tasks and leads to the rapid development of human history toward the information age. By now, von Neumann architecture-based computers have shown ubiquitous applications in many fields like image processing, data mining, and so forth. But the challenging issues, including the time consumption and the energy expense of the mainstream computing architecture, are growing due to the arrival of the Big Data Era. On the one hand, the raw data to be processed is becoming extremely large. For example, open-source data sets like Open Images [2] and ImageNet [3] all have hundreds of GB of data. On the other hand, the parameters for the ML tasks, especially the ANNs, are increasing dramatically. Mainstream benchmark models such as VGG [4] and ResNet [5] models possess millions of parameters. The separated structure of the CPUs and memories in the hierarchy architecture makes it costly for frequent data communication since the data are fetched serially from the memory, which is also known as the “von Neumann bottleneck”. Even though, the emerging of the graph processing units (GPUs) has obtained great accelerations for large data processing and eased the limitation of the speed mismatch by parallelly processing the structured data and lowering the distance for the data transmission [6, 7]. But the physically limited constraints still exist in the separated structure, and a revolutionary change in the computation architecture is required to break the von Neumann bottleneck.

One of the most straightforward ideas is to functionally and physically merge the data processing and the memory storage unit, which we now prefer to call it the in-memory computing (IMC) paradigm [8]. As the name suggests, IMC architecture processes the data at the locations where they are stored and performs the calculation in the memory block in an in situ manner. Therefore, in the IMC architecture, frequent data communication can be avoided to reduce the time delay and the corresponding energy consumption. The demand for IMC has further promoted the growth of the emerging non-volatile memory (NVM) devices. Memory devices are served as the core component of IMC. Compared to the volatile memories such as static random-access memory (SRAM) and dynamic random-access memory (DRAM), the NVMs utilize the non-volatile conductance to store the data, which could be operated by external voltage stimuli directly and maintain the status even after the voltage removal. Organized in the crossbar array, the NVMs show highly parallelism to execute the matrix–vector multiplication (MVM) in a single step regardless of the dimensions of the vectors.

**Fig. 1:**
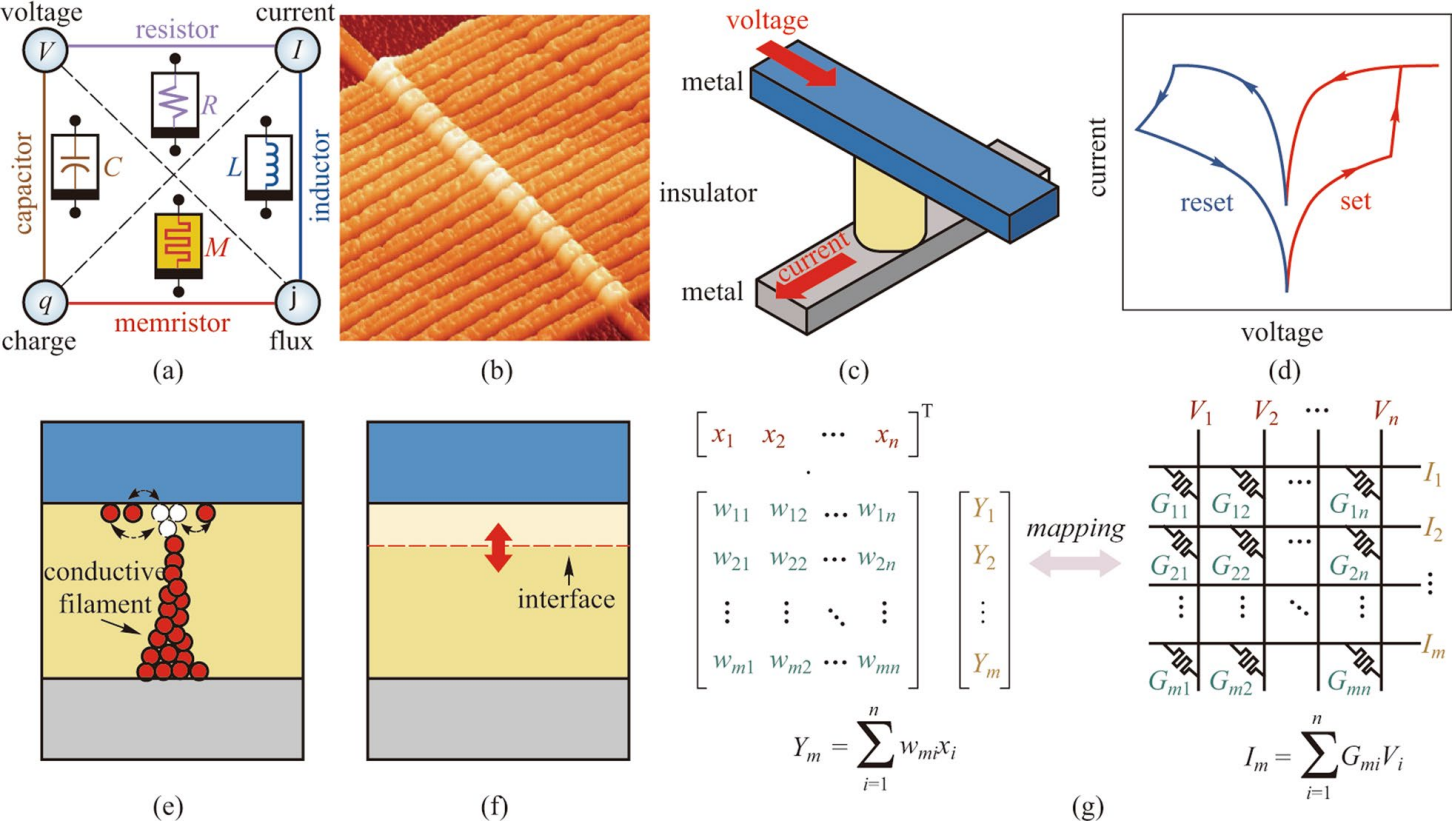
Schematics of memristors and memristive in-memory computing. **a** Four fundamental passive circuit elements and their relationships. The memristor is defined by a relationship between flux (*φ*) and charge (*q*). Adapted from Ref. [9]. **b** A scanning tunneling microscope image of memristor array. Adapted from Ref. [10]. **c** Illustration of a memristor with a metal/insulator/metal sandwiched structure. **d** Typical *I*–*V* curve of a memristor shows a hysteresis loop under the voltage sweeping, indicating a reversible resistance modulation capability. Schemes of the conductance tuning mechanisms of **e** conductive-filament type and **f** interface type memristors. **g** Schematics of the matrix–vector multiplication (MVM) operation mapping into a memristor crossbar array by exploiting Ohm’s law and Kirchhoff’s law

Memristor was first theorized by Chua in 1971 as the fourth fundamental passive circuit element in addition to the resistor, capacitor, and inductor (Fig. 1a) [9, 11]. Since the first experimental demonstration of the TiO_2_ devices by HP laboratories in 2008 (Fig. 1b) [10, 12], memristors have received great attention as a promising candidate of the emerging NVMs. Memristors usually possess an extremely simple structure with one or several insulator functional layers sandwiched between two metal electrodes (Fig. 1c), leading to high potential for integration, scaling down, as well as 3D stacking [13–15]. According to the different conductance tuning mechanisms, various memristors can be mainly divided into the conductive-filament type that relies on the formation and decomposition of the conductive paths across the functional layers (Fig. 1e), and the interface type that modulates the thickness of the functional layers (Fig. 1f). These versatile mechanisms make the conductance tuning of the memristor cells adjustable, and provide two distinct routes for memristive IMC implementation. As shown in Fig. 1d, basically, the binary resistive switching behaviors of the memristors with on/off states, which are usually achieved by the abrupt formation/disruption of the conductive filaments, are used to map the binary 0/1 values. Such behaviors not only can be used for nonvolatile data storage but also is capable of implementing Boolean logic computation. The logical completeness utilizing the binary memristors has been confirmed in previous studies, which has been proposed to be applied in nonvolatile processors [16], encryption electronics [17], high-dimensional computing [18]. Especially, the XOR logic in the memristors has been used for the ternary content-addressable memory (TCAM) design with much less area and higher efficiency than CMOS technology [19, 20].

Furthermore, by gradually manipulating the formation of the conductive filaments or the thickness of the functional layers, memristors show the capability of multilevel modulation, which provides multilevel or analog conductance states in one cell. Extensive studies have revealed that the state-of-the-art memristors can reach over 128 distinguishable states, and achieve higher storage density and data processing efficiency compared to the binary ones [21, 22]. The analog memristors have demonstrated prominent potential as the core component of IMC to support various analog computing applications including ANN, ML, scientific computing, digital image processing, and so forth, seeking consecutive research efforts [23–27].

In this review, we conduct a thorough survey on the recent advanced progress of memristive in-memory computing. This paper is organized as follows. Section 1 reviews the background of in-memory computing and the basic characteristics of memristors as the core component. Section 2 introduces the principles of memristive in-memory computing paradigm, and presents the categorization criteria of the application fields into soft and hard computing types. Section 3 mainly focuses on various machine learning accelerators as representative soft computing applications. Section 4 focuses on memristive scientific computing and digital image processing on behalf of the hard computing domain. Finally, in Sect. 5 we conduct a brief conclusion of this paper, discuss the remaining open challenges, and envisage further development of the memristive in-memory computing paradigm. For this review, we hope it will attract rising attention and further inspire substantial researches in this novel field.

## Memristive in-memory computing

The prominent acceleration of memristive IMC mainly falls on the high parallelism of executing MVM using the memory arrays. As one of the most fundamental and essential mathematical operations, MVM is the core step of many applications like digital signal processing, ML algorithms, and equation solving, etc. In traditional processors like CPUs and GPUs, MVM operations are usually performed by a set of dedicated hardware units called multiply-accumulate (MAC) units. A MAC unit is composed of a multiplier followed by an adder and a register that stores the partial accumulative results. In each calculation cycle, the data fetched from the external memories are multiplied, and the output is accumulated to the register. However, such processes involve continuous-time consumption, register operations, and data accesses according to the processed matrix size.

Comparatively, the memristor crossbar array possesses a fully connected topology, enabling high parallelism of MVM processing, as shown in Fig. 1g. In the memristor array, the input vector is encoded as the time-serial input voltage vector, and the multiplied matrix is encoded as the conductance of the memristors. Through Ohm’s law and Kirchhoff’s current law, the output vector can be directly obtained from the output current of the memristor array conducting MVM operations in *O*(1) time complexity regardless of the matrix size. Therefore, utilizing memristive IMC has become a promising substitution to efficiently accelerate the MVM operations and the various corresponding applications. As a computing paradigm located in the analog computing domain, the accuracies of the calculation results of memristive IMC methods are directly restricted by the hardware characteristics. On the one hand, the limited conductance states of the devices will cause quantization errors into the mapped matrix. In addition to that, the device variation due to intrinsic stochasticity will introduce deviations between the mapped matrix and the ideal one. On the other hand, the precision of the periphery circuits, mainly the digital-to-analog converters (DACs) and the analog-to-digital converters (ADCs), is also limited. DACs are used to generate input voltage signals with different amplitudes according to the vector. In particular, other input methodologies have also been proposed to encode the input vectors into binary input pulse sequences or width modulated pulses according to the input precision [28]. ADCs are utilized to sample the output signals and generate the results of MVM. Ideally, to conduct the conversion of the results with full precision, the precision of the ADC should satisfy the relationship defined as ${b}_{\mathrm{ADC}}={b}_{\mathrm{in}}+{b}_{\mathrm{d}}+{\mathrm{log}}_{2}N$, where ${b}_{\mathrm{in}}$ and ${b}_{\mathrm{d}}$ is the precision of the inputs and the devices, and $N$ represents the accumulation number. However, such high-precision ADCs require ultra-high hardware overhead, which is usually difficult to meet, especially when the accumulation number is large. Therefore, all of these factors will inevitably introduce quantization errors during the signal conversions.

In Table 1, we summarize a few representative experimental demonstrations of IMC using memristor arrays for different practical applications. It is notable that other mainstream NVM-based IMC have also been widely investigated, such as flash [29], phase change memory (PCM) [30], ferroelectric field effect transistor (FeFET) [31], magnetic random-access memory (MRAM) [32], and so on. Although the memristive IMC paradigm is validated in such widespread domains, the different applications claim distinct requirements on the computational accuracies and the corresponding hardware solutions including both memory devices and periphery circuits [33–35]. For instance, scientific computing applications usually require ultra-high precisions since even relatively small errors in the intermediate results can lead to outright mistakes, and emphasize the accuracy of the numerical result. “Hard” requirements on the computational accuracies with effective compensation strategies should be satisfied for such “hard” applications. On the contrary, neural network inference applications can be conducted in much lower precisions, and focus more on the inherent and relative associations of data rather than the specific numerical values, which make them among the typical “soft” applications, and greatly ease the hardware design demands [36]. Therefore, we roughly divide the wild range of the different applications into soft and hard computing according to their different demands on the computational accuracies, and organize the following review in this framework.

**Table 1:** Summary of the representative experimentally demonstrated memristor arrays

Array type	Memristor material	Array size	Conductance states	Application demonstrations	Performance /(TOPS⋅W^−1^)	References
1T1R	Ti/HfO_2_/TiN	128 × 64 8 Kb	2	BNN	24.1	[37]
1T1R	TiN/TaO_*x*_/HfO_*x*_/TiN	512 × 1024 0.5 Mb	2	BNN	−	[38]
1T1R	W/TiN/TiON	2 Mb	2	ANN	146.21 (binary) 36.61 (multibit)	[39]
R	TiN/Al/Ti/TiO_2−*x*_/Al_2_O_3_/TiN/Al/Ti	64 × 64 4 Kb	~ 32	MLP, combinatorial optimization	81.33	[40]
1T1R	TiN/TaO_*x*_/HfO_*x*_/TiN	2 Kb	32	CNN	11	[41]
1T1R	TiN/TaO_*x*_/HfAlO/TiN	1 Kb	~ 500	MLP, on-chip training	−	[22]
1T1R	Ta/HfO_2_/Pt	128 × 64 8 Kb	100	MLP, LSTM, RL, on-chip training	77.4	[42–45]
R	Pd/WO_*x*_/Au	54 × 108 5.7 Kb	100	MLP, on-chip training, sparse coding, PCA	0.188	[46, 47]
R	Ta/Ta_2_O_5−*x*_/Pd	16 × 3	2	PDE solving	60.1	[48]
1T1R	Ta/HfO_2_/Pd	128 × 64 8 Kb	128	MLP, signal processing, image compression, convolutional filtering	199.7	[21, 49]

Application demonstration name abbreviation: *BNN* binary neural network, *CNN* convolutional neural network, *MLP* multi-layer perceptron, *LSTM* long short-term memory network, *RL* reinforcement learning, *PCA* principal component analysis, *PDE* partial differential equation, *TOPS/W* Tera operations per second per watt

## Soft computing

Soft computing refers to the tasks that can be processed with relatively low computational accuracies and tolerate uncertain and imprecise results. ML is one of the most representative and important domains of soft computing. ML algorithms usually concentrate on learning a specific model from the inherent and relative associations of the data, and seek to make classification, clustering, or regression to it. The generalization capability required by a good ML algorithm postulates that the learned model should focus on the overall accuracies and tolerate the noises of each data and the relevant result, leading to the great relief of the hardware cost on the computational accuracies. Following the good compatibility of the memristive IMC and soft computing, the hardware implementations of ML algorithms have fetched a great stride of progress. In this section, the memristive ANNs are first discussed with both the inference and training phases. Then the non-ANN ML tasks which utilize the memristor arrays to ease the pressure of the curse of dimensionality are introduced. Finally, we discussed some special ML applications that employ the inherent stochasticity of the memristors.

### Memristive artificial neural network

Artificial neural networks are inspired by the biological neural networks of human brains, which can adaptively implement various models through their connected structure [50]. ANNs have brought significant breakthroughs for ML and become the mainstream solution for various applications like natural language processing [51], image classification [52], and object detection [53]. However, the powerful representation capabilities of the ANNs come at the cost of the increasing volume of the network parameters and the calculation procedures [54]. Their applications are gravely restricted by the high intensities in both data storage and movement, especially when computing resources are highly restricted for edge intelligence and Internet of Things (IoT) applications. Under these considerations, utilizing memristor arrays to accelerate ANNs has become one of the most promising solutions and has attracted extensive interest. Memristive neural networks involve two major application phases, inference and training. In this section, we will introduce several representative works on the hardware implementation of the memristive neural networks, mostly based on the inference applications, and conduct a thorough review of the different approaches of the training phase.

#### Memristive neural network inference

Memristive neural network inference applications can also be referred to as the neural network accelerators and the off-line training method. In these cases, the networks are well trained on software and transferred to the array as the memristor conductance, while only read operations are conducted on the hardware without involving complicated and frequent operations to update the memristors. Thus, such a method can take full advantage of the non-volatile characteristic of the memristors and eliminate frequent device update requirements, which makes them more convenient to be implemented.

**Fig. 2:**
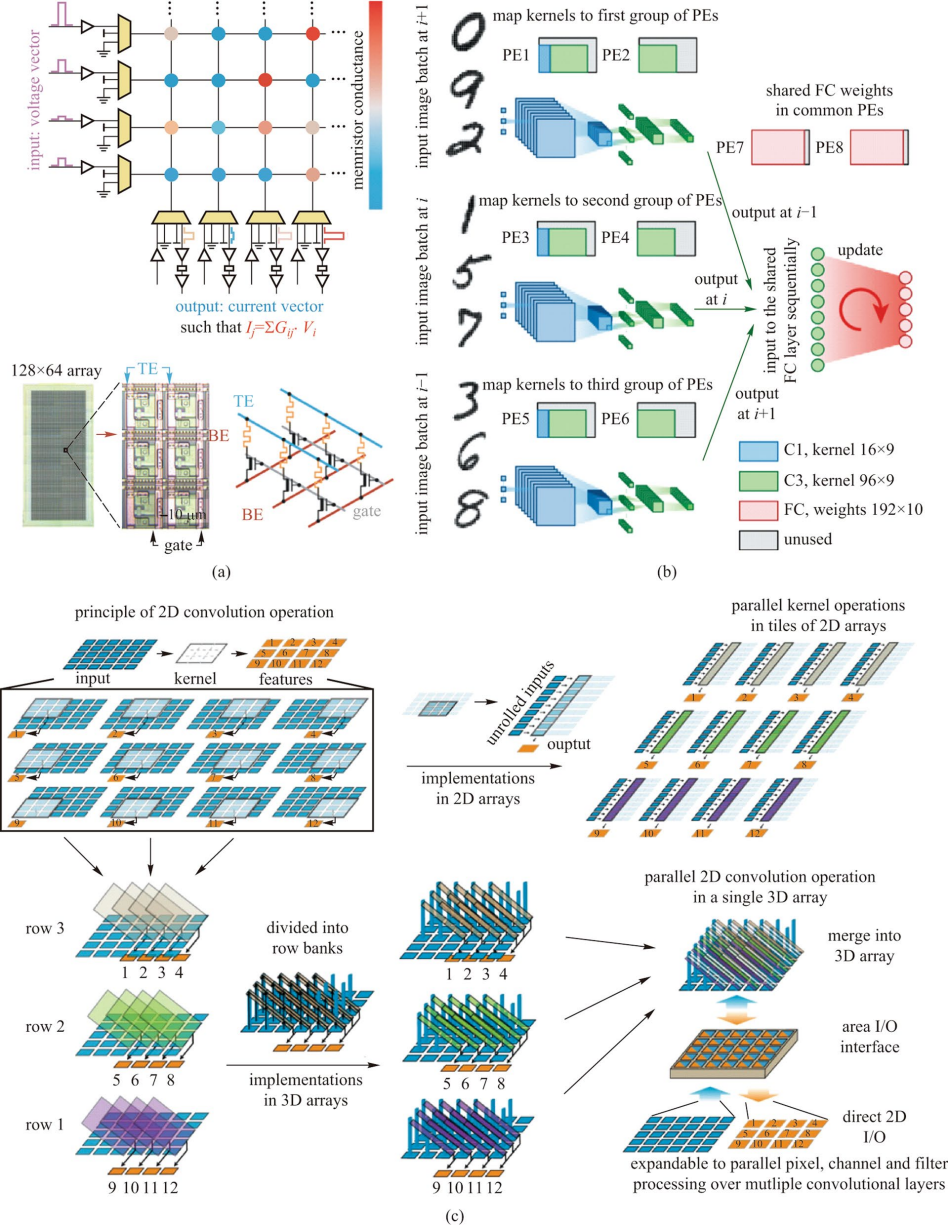
Schematics of the hardware-implemented memristive neural network. **a** MVM scheme with memristor arrays and the periphery circuits using the fabricated 128 × 64 1T1R array. Adapted from Ref. [21]. **b** Sketch of the hardware system operation flow with the duplicated convolutional weights and the hybrid training method. Adapted from Ref. [41]. **c** Schematic of the mapping strategy of the convolutional operations in the 3D memristor array with customized row bank structures. Compared to 2D arrays where full rearrangement of the weight kernels and the inputs are required to process convolutional operations, the demonstrated 3D arrays only need a few shift and replicate operations. Adapted from Ref. [56]

MLP is regarded as the most fundamental type of ANN. It consists of several fully connected layers, which generally conduct an MVM operation on the inputs and the weights, and can be easily transferred to the memristor arrays. Besides that, the fully connected layers are also frequently used in other complex models. The simple structure makes MLP the most popular model for memristive neural network prototype demonstrations in their early stage [22, 55], and two representative studies are reviewed in detail. In 2018, Hu et al. demonstrated an analog 128 × 64 1T1R array to implement a single-layer neural network for handwritten digit classification, as demonstrated in Fig. 2a [21]. Depending on the great controllability of the transistors, 6-bit precise analog conductance tuning can be achieved. DAC arrays were applied to generate voltages as input signals. Transimpedance amplifiers (TIAs) and ADC arrays are applied to convert output currents to voltages and sample the output voltage signals as the final result of the MVM operation, respectively. Thus, a computational precision equivalent of 6 bits was achieved, and a classification accuracy of 89.9% was acquired on the MNIST data set. Although the utilization of DACs and ADCs will introduce a heavy hardware overhead in area and energy consumption, it enables the interconversion of signals between the analog domain and the digital domain. Therefore, the memristive neural networks can take advantage of not only the high parallelism and energy efficiency of the MAC operations on the memristor arrays but also the high flexibility and re-programmability of other essential operations in the digital processors. Such strategy provides an essential compromise between generality and performance enhancement and has become the most primary method for computing in memristor array.

Recently, Kim et al. proposed an impressive demonstration of MLP on a 64 × 64 passive memristor crossbar array [40]. Passive arrays possess higher integration density and an easier fabrication process than the 1T1R arrays. But the lack of transistors means that the programming pulses of the other memristors will influence the already tuned devices, making it much harder to accurately program the conductance. To program the memristors more precisely, they conducted a thorough statistic of the target conductance with respect to the initial state and the programming pulses over the fabricated 4096 devices, and extracted the tendency through fitting to guide the conductance tuning. In addition, to address the crosstalk problem, a half-biasing scheme was adopted, and the tuning of the whole array was repeated several times to compensate for the disturbance. Through these optimization methods, an average relative tuning error of < 4% can be achieved for about 32 states.

With increasing data volume and complexity, MLP is no longer capable of solving real-world tasks, especially for image processing problems. In such circumstances, CNN is proposed. Compared to MLP, the most significant difference of CNN is that it introduces the convolutional layers for feature extraction, which are greatly inspired by the behaviors of bio-vision systems. Benefitting from the convolutional layers, CNN cannot only process 2D or 3D information directly with high robustness, but also greatly ease the number of required parameters through the weight sharing method. Other than the convolutional layers, a typical CNN also consists of pooling layers and fully connected layers, which are used for down-sampling and classifying, respectively. In the convolutional layers, the weight kernels are slid over and convolved with the input feature maps. Since that, unlike the fully-connected layers, the convolutional layers cannot be directly transferred to the memristor arrays. The common solution is to divide the whole input images into time-serial partial input ones according to the process of convolution operations, and rearrange both the partial input images and the weight kernels into 1D vectors. Therefore, extra periphery circuit designs are required for controlling, rearranging, and buffering the intermediate data, which makes CNN much more complicated to be hardware-implemented.

In 2020, Yao et al. proposed a hardware-implemented memristive CNN on the fabricated and integrated eight 128 × 16 1T1R chips, as illustrated in Fig. 2b [41]. The evaluated CNN model was composed of two convolutional layers and one fully-connected layer. To conduct MVM operation, rather than using the DACs, the input signals were encoded as sequences of pulse numbers, while shift-and-add operations were required to generate the final output result. To compensate for the mismatch of the processing speed between the convolutional layers and the fully connected layers, they duplicated the weights of the convolutional layers into three independent memristor arrays for parallel processing. Besides that, to overcome the transferring difference between the software calculated weights and the memristor conductance, the hybrid training method was introduced by updating the weights of the final fully-connected layer after the weight transferring. Therefore, the nonidealities of the memristors can be partially overcome at small efforts. Based on the hardware-implemented memristive CNN, energy efficiency of 11 TOPS/W can be obtained, which is 110 times better compared to the Tesla V100 GPU.

In the same year, Lin et al. proposed a customized eight-layer 3D memristor array for the experimental demonstration of hardware-implemented CNN, as shown in Fig. 2c [56]. In the customized 3D arrays, the input pillar electrodes and the output staircase electrodes provide electrical isolation between the vertical memristors, while still maintaining the high density of the 3D stack structure. Thus, it can parallelly process those convolution operations that would otherwise be temporal in 2D-array solutions, and can also greatly suppress the array size effect like sneak path current and IR drop. Such modified memristor array structures are promising candidates for memristive CNN accelerators, especially in edge intelligence applications.

Apart from the MLPs and CNNs, many other kinds of memristive neural networks have been investigated and validated to extend the capability for various applications [42, 43, 57, 58]. For instance, Li et al. illustrated the implementation of long short-term memory (LSTM) networks on the memristor crossbar system, which are capable of processing time-serial signals such as videos and language translation [44]. Li et al. proposed a demonstration of the memory-augment neural networks (MANN) on a 64-Kb memristor memory, enabling the learning of unseen classes with only a few samples and overcoming the shortcomings of requiring large training data in the traditional neural networks [59].

#### Memristive neural network training

As described above, in inference applications, the parameters of the neural networks are well-trained on the software, and then transferred to the memristor arrays. Such a method ensures that only one time of conductance programming is needed, easing the requirement of the memristor characteristics and the periphery circuit design, but it is not flexible for practical applications. Under these considerations, memristive neural network training, also termed online/on-chip training, is receiving progressive attention as one of the critical future application formats. Memristive neural network training involves the update of a certain part of the network weights, which provides two main advantages. One is that it makes the networks possible to accommodate the nonidealities of the memristors, e.g., device-to-device variation, device failure, and yield. The other is that the flexibility and universality of the network are enhanced for various practical applications. However, online training in turn brings high demands on the strategy design and the hardware mapping method.

The online training of the neural networks consists of two major phases, the forward propagation to calculate the outputs and the backpropagation to process the errors and the parameter update values. As been discussed above, the forward propagation can be straight-forwardly mapped to the memristor arrays just like the inference process. The challenging issue focuses on the realization of the error backpropagation process during the training iterations. The error backpropagation (BP) is the most outstanding learning algorithm to enable the mighty functionalities of the neural networks. However, mapping BP to the memristor arrays is much more complicated, and various kinds of approaches have been proposed, which provide the guidelines for online training. The easiest solution is to use the memristor arrays for forward propagation while the software for BP, which ensures the high accuracy of the calculations [22, 42, 45]. However, the required high-precision digital processors are usually inaccessible in practical application circumstances. A promising substitute solution is to use the limited-precision, i.e., no more than 16-bit, or fixed-point digital processors instead, which requires discrete training techniques to compensate for the reduced training precision. For instance, Wu et al. proposed a discrete training method termed WAGE using binary for inference and 8-bit for BP, and validated on the VGG benchmark model [60]. Zhang et al. described a sign BP method with binary inference and 2-bit BP for the training of MLP [61].

In the BP process, the propagation of the errors layer-by-layer involves the multiplication of the transposed weight matrix. Researches have also been conducted to accelerate the processing of these steps in the memristor arrays by converting the input directions [61–63]. The acceleration of fully connected layers is rather straightforward and easy to transfer onto memristor arrays [46, 64]. But that of the convolutional layers is on the contrary very complex. It involves the convolution operation of the error matrices and the transposed weight matrixes, which requires extra data rearranging efforts, and yet lacks experimental demonstrations. An impressive work proposed by Jiang et al. demonstrated a MINT method for in-memory training with mixed-precision on the 1T1R memristor arrays [65]. To take advantage of the stable characteristics of the binary memristors while achieving high-precision analog computing, multiple devices with multipliers and adder trees are used. The memristor arrays mapped as the weights are divided into 2 tiles representing the most significant bits (MSBs) and 6 tiles representing the least significant bits (LSBs). The processing of the forward and the BP are conducted on the arrays for MSBs only, while the weight update is performed and accumulated on the arrays for LSBs to ensure the training accuracy. The proposed architecture can obtain an estimated energy efficiency of around 4.46 TOPS/W.

Apart from the methods described above, many other strategies have also been proposed to ease the hardware implementation of memristive neural network learning. Negrov et al. described an approximated BP algorithm in an MLP by encoding the calculation of the delta values into different polarities of update voltage pulses [66]. Yao et al. proposed the hybrid training method, as have been described above, by conducting on-chip learning only on the final fully-connected layer of the CNN, achieving effective suppression of the device non-idealities while avoiding updating the entire network [41]. Lu et al. utilized a newly proposed training algorithm named the direct feedback alignment (DFA) [67] that conducts the errors directly into each hidden layer instead of BP that conducts the layer-by-layer error propagation to accelerate the on-chip training [68]. The randomness of the PCM arrays was used to physically generate the fixed random feedback matrices, which are of vital importance in the DFA. The results show that compared to hardware-accelerated in-memory BP training, 3 × and 3.3 × of the time and energy consumption can be saved.

Present researches have provided excellent demonstrations of the memristive neural networks on enhancing the energy efficiency and the processing speed in both inference and learning applications. Apart from the different mapping strategies of the two applications described above, another critical aspect is the distinct requirements on the device characteristics. In inference applications, the accurate programming of the quantized conductance states is the top concern, and write-verify strategies are usually applied to reduce the variations of the conductance states [22, 40, 69, 70]. Researches have also spotlighted lowering the precision of the inference process to ease the hardware burden through quantization strategies, e.g., 8 bit for industry and lower for academia [71–73]. As for the learning applications, it is well recognized that high precision and stability of the weight update are extremely critical during the network training, demanding large numbers of tunable conductance states, low cycle-to-cycle variation, as well as high symmetricity and linearity in the potentiation and suppression [74, 75]. Various studies have been conducted to optimize these critical analog tuning behaviors of the memristors through material modification and programming method optimization [75–77].

### Non-ANN based memristive machine learning algorithms

Although ANNs have taken full advantage of accelerating MVM and made great achievements during both the forward and backward processes, the sharp increase of data dimensions still lies on the road ahead of the efficient processing of ML algorithms. The curse of dimensionality, which indicates the problems such as sparse data and difficulty in distance calculation of high-dimensional data, has hindered the future development of ML tasks. Solutions to the curse of dimensionality could be dimension ignorable computation or efficient dimension reduction methods. The constant time complexity of MVM operations in the IMC paradigm makes it possible to handle hyperdimensional data with much faster speed. Unlike basic ANN applications where the memristor arrays can be utilized directly, ML tasks rely much more on the specified data mapping methods or approximate calculation strategies to deal with complex operations during the data processing. Studies have proved that memristor arrays perform well in the acceleration of the various ML tasks. In this part, we review the studies that focus on the memristor-accelerated ML tasks and introduce several typical works that have made significant progress to ease the curse of dimensionality.

Serving as one of the most essential parts, similarities not only can be commonly used in both the data analysis and data searching fields, but also are the hardest-hit area that suffers from the curse of dimensionality. The commonly used similarities like Euclidean distance (ED), cosine similarity, are directly employed in the analog computing domain for the algorithms such as K-means data clustering, nearest neighbor search, self-organized map, etc. For the primary demonstration of the similarity calculation in the memristor crossbar array, ED, the most generally used distance description method in Euclidean space, is first and wildly studied for acceleration on the memristor arrays. The ED of the vectors *U* and *W* is determined by $\parallel U-W\parallel ={U}^{2}-2U\cdot W+{W}^{2}$. Other than the dot product process, the squared terms are also necessary for accurate calculation. But these terms show less friendship for the hardware implementation and acceleration on the memristor crossbar array. Studies to accelerate the ED calculation in the memristor mainly focus on solving these terms on hardware. Yu utilized the dot product results calculated from the RRAM array to approximately represent the ED, and achieved a winner-take-all neural network for orientation classification function [78]. Such a method seems rough for the distance calculation, but is still partly valid for the self-organized map application as evaluated in the work. More importantly, the work brings the concept of similarity measurement to the crossbar array for the first time. However, such dot-product representation simplifies the ED calculation so much that it becomes inaccurate for general data comparison. Under these considerations, Jiang et al. proposed a specialized mapping method in the RRAM to implement the fully ED and the k-nearest neighbor classification algorithm, which pays much more attention to the inference, and was realized with high performance [79].

**Fig. 3:**
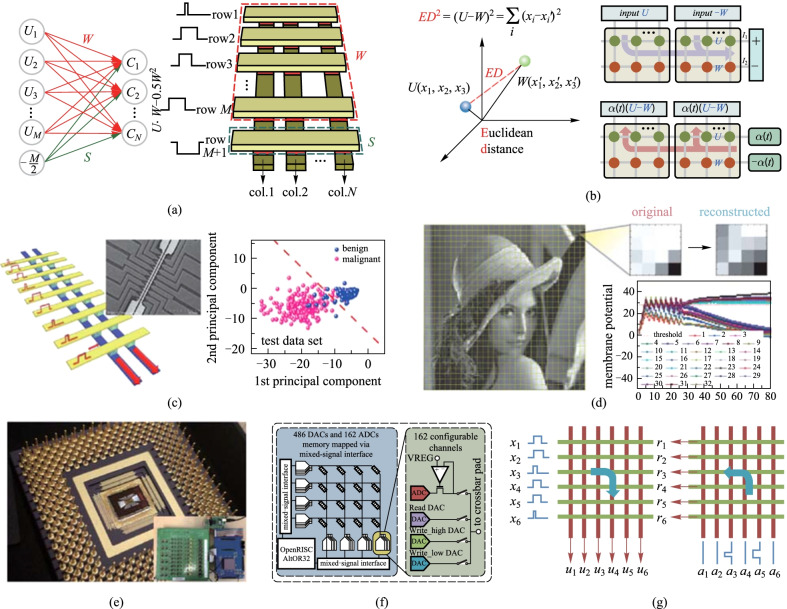
Machine learning algorithms on memristor arrays. **a** K-means data clustering by calculating $-2U\cdot {W}_{n}+{S}_{n}$ for data comparison. Adapted from Ref. [80]. **b** Mapping method and operation steps of the Euclidean distance engine for competitive learning. Adapted from Ref. [81]. **c** Implementation of PCA to the memristor arrays and the experimental verification with the Breast Cancer data set. Adapted from Ref. [82]. **d** Demonstration of the sparse coding. Adapted from Ref. [47]. A general programmable solution for various ML algorithms implemented by **e** fully integrated memristor chip with **f** configurable ADCs and DACs both in rows and columns, enabling **g** bi-directional data flow. Adapted from Ref. [46]

Following that, Jeong et al. realized a fully hardware-based ED calculation method to achieve the K-means data clustering algorithm, which is widely utilized in various applications, as shown in Fig. 3a [80]. K-means data clustering aims to find the most representative prototype vectors of the classes by measuring the Euclidean distance between the input vector *U* and the weight vector *W*_*n*_, where *U* is mapped as the input signals, and *W*_*n*_ are stored in the array. During the distance comparison, the ${U}^{2}$ term is constant for all stored weight vectors and can be directly ignored. By adding an additional row to the RRAM crossbar array to store the ${{S}_{n}=W}_{n}^{2}$ term, the parallel MVM can realize the distance calculation determined by $-2U\cdot {W}_{n}+{S}_{n}$ in one step. For the training process of clustering, the learning rule is determined by $\Delta W=\eta (\mathrm{input} - {W}_{n})$ and $\Delta {S}_{n}=\sum_{j}{W}_{jn}^{2}-{S}_{n}$. With the assistance of the digital processors, such implementation provides a solution for the training of the adaptive weights during the clustering. The complete algorithm was then experimentally achieved in the RRAM array using the IRIS data set, and obtained comparable accuracy with the software-based solutions.

Adhere to the concepts in Refs. [79] and [80], a recent study accomplished by Zhou et al. proposed an energy-efficient ED engine by reshaping the ED calculation to $U \cdot \left(U-W\right)+(-W) \cdot (U-W)$ and mapping it to the crossbar array, as demonstrated in Fig. 3b [81]. Using the differential structure of the memristor array, the ED engine was experimentally demonstrated in the 1T1R array with constant temporal complexity. The structure of the ED engine ensures the hardware-dependent data update method for competitive learning and reduces the complexity of the periphery circuits. The ED engine was then used for both online training and offline inference for competitive learning and achieved times of improvement in energy efficiency compared to the GPU platform. These studies have made considerable progress for ED-based applications, but the cosine similarity, which has greater advantages in processing sparse vectors, still remains uncovered. The main challenge in accelerating the cosine similarities in the crossbar array lies in the L2-normalization part. Zhou et al. first developed a cosine similarity calculation method based on the spherical data utilizing the RRAM arrays, and proposed an online training method for cosine similarity-based K-means data clustering [83]. The simulation results show its reliable capabilities in text classification and further data analysis for sparse vectors.

Another way to handle the problems of high-dimensional data are to efficiently extract the features and reduce the dimensionality of the original data. A typical method to extract the features of the data are the PCA algorithm, which projects the original data to the uncorrelated vectors in a new space. The uncorrelated vectors are called the components, and the ones that matter most are selected as the dimensionally reduced data. Choi et al. utilized the memristor crossbar array to accelerate the compute-intensive feature selection process in PCA with the unsupervised online learning method, as illustrated in Fig. 3c [82]. The PCA method, which traditionally relies on solving the covariance matrix, is achieved by Sanger’s rule in an online learning manner, and the updating process is determined by

1 $$\Delta {g}_{ij}=\eta {y}_{i}\left({x}_{i}-\sum_{k=1}^{j}{g}_{ik}{y}_{k}\right),$$

where *x* and *y* denote the input vector and the output vector respectively, ${g}_{ij}$ is the weight at row *i* and column *j* in the neural network and stored in the array, $\eta$ is the learning rate. The performance of the PCA network is then experimentally proved in memristor array and the extracted data achieve the reliable data classification and prediction tasks. At the same period, Sheridan et al. proposed a sparse coding method on the memristor arrays, as shown in Fig. 3d, which utilized a series of dictionary elements to represent the original data [47]. The crossbar arrays were employed to store the dictionary elements, and the representative vector was defined by the linear combination of the stored features. The coefficients of the combination exhibit the characteristics of sparse vectors, which means that the original signal can be represented by a few dictionary elements with lower data dimensionality. Experimental demonstration on a 32 × 32 crossbar array was implemented and the reliability of sparse coding was verified for the applications like computer vision, signal processing, etc.

From the view of the memristor crossbar arrays, a reconfigurable memristor system is recommended for these various ML algorithms with different mapping methodologies. Guided by this mind, Cai et al. proposed a fully integrated memristor-CMOS system to implement different MVM-intensive algorithms [46]. The passive WO_*x*_-based memristor array with indispensable peripheral circuits including the DACs and ADCs, the interface circuitry, digital buses, and the programmable processors are all fabricated in a whole system (Fig. 3e). Especially, both the rows and the columns provide configurable ADCs and DACs which made it flexible for the mixed-signal communication between the array and the processor, although this may increase the area of the chip (Fig. 3f). Such design enables bi-directional data flow (from the rows to the columns and the columns to the rows, as shown in Fig. 3g), and makes it possible to implement both the forward and the backward read processes, which can be utilized in the inference and the backpropagation of the neural networks. Applications including the MLP, PCA, and sparse coding were also demonstrated, providing the potential of a general programmable solution for the ML hardware accelerating system.

### Stochasticity and optimization problems

Available multilevel conductance is the basis of memristor-based analog ML tasks. But the inherent stochasticity of the conducting mechanism and the changes of the conductive filaments inside the memristor devices, make the conductance states unstable and noisy, and vary from the desired values. Although the previously stated soft computing tasks can tolerate moderate degrees of noise, high stochasticity of the conductance is still undesirable, which could lead to disappointing results, and should be avoided through optimized programming schemes [69, 84]. However, it is interesting to note that the noisy conductance states demonstrate high randomness with Gaussian distributions [85, 86]. Such stochastic distribution is considered possible to be implemented in various probability-based algorithms.

**Fig. 4:**
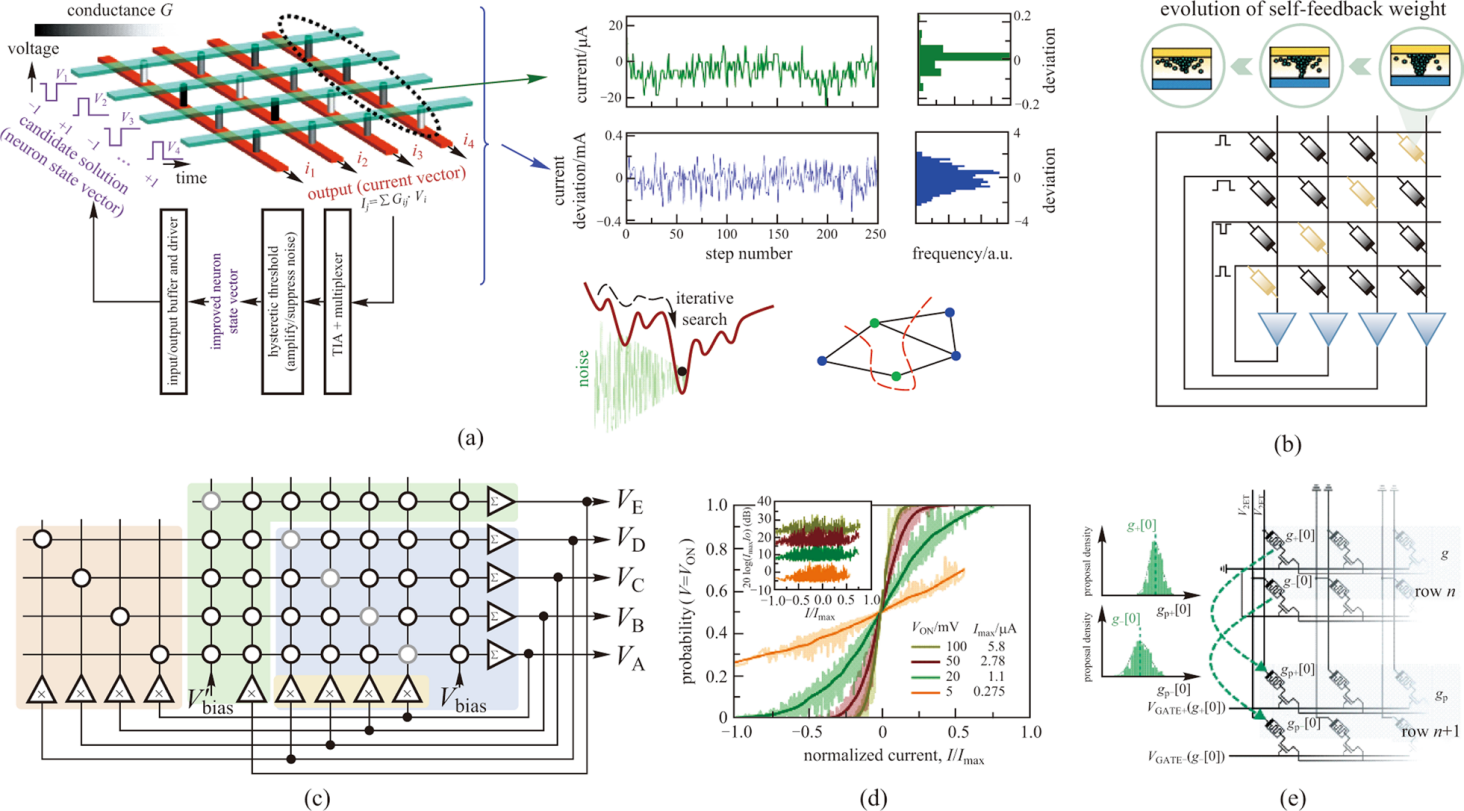
Concepts of solving optimization problems with the stochasticity of the devices. **a** Schematic of memristive HNN optimized by periphery circuit modulated intrinsic noises. Adapted from Ref. [88]. **b** Mapping strategy of the hardware-implemented TCNN using diagonal memristors as the self-feedback weights. Adapted from Ref. [89]. **c** A versatile stochastic dot product circuit for the implementation of generalized HNN. The blue, yellow, green, and red backgrounds highlight the implementation of basic HNN and stochastic, adjustable, and chaotic approaches, respectively. The circuit modules labeled with Σ/ × denote summation/scaling operations. **d** An example of the measured stochastic neuron transfer functions at several applied voltages, i.e., different effective computing temperatures, for the hidden neuron. The inset shows peak signal-to-noise ratios. Adapted from Ref. [90]. **e** Evolution strategies of the MCMC sampling method. Adapted from Ref. [91]

An extensively employed application that can take advantage of the randomness of the memristors is the combinatorial optimization task where the Hopfield network (HNN) serves as one of the common approaches [87]. The HNN can solve such optimization problems by converting the task objective into its energy function, and minimizing its energy through iterations. However, HNN can easily converge to local minima and result in unsatisfactory solutions. To address this problem and further improve the energy efficiency, Cai et al. proposed an impressive demonstration of the memristor HNN and utilize the intrinsic noise of the memristor devices for optimization, as demonstrated in Fig. 4a [88]. For a given task, i.e., the max-cut problem as demonstrated in the work, the weight matrix predetermined by the task is transferred to the memristor array, and the binary neuron states are encoded as the voltage pulse amplitudes. To prevent the network from being trapped by local minima, they utilized the intrinsic noise of the memristor devices and designed a hysteretic threshold circuit to achieve noise modulation. The designed circuit was first adjusted to amplify the intrinsic noises at the beginning of the network iteration to jump out of local minima, and then suppress the noise for network convergence. Simulations suggested that over four orders of magnitude higher energy efficiency can be obtained compared to other approaches. But the complex sequential periphery circuits still require extra design efforts and hardware overhead.

Yang et al. demonstrated a variant of the HNN termed as transiently chaotic neural network (TCNN) on the fabricated memristor array for Max-cut problems, as illustrated in Fig. 4b [89]. Compared to vanilla HNN, TCNN possesses a temporal self-feedback coefficient to modulate the network from dispersion to convergence to skip the local minima [92]. To efficiently implement the network and achieve the modulation of the self-feedback coefficient, they mapped the weight of the self-feedback coefficient as the conductance of the memristors on the diagonal of the array, and conduct the modulation through the evolution of the conductance. Such a strategy can achieve highly compact memristive HNN hardware implementation, but the frequent programming of the diagonal memristors, in turn, becomes an inevitable burden. Apart from these researches, Lu et al. quantitatively evaluated the effect of memristor read noises to optimize the HNN for traveling salesman problems [93]. Fahimi et al. proposed a weight annealing approach for HNN on a fabricated 20 × 20 analog memristor array with the help of the designed mixed-signal circuits [94]. A more versatile stochastic dot product circuit was proposed by Mahmoodi et al. for generalized HNN, which could implement stochastic, adjustable, and chaotic approaches in the same circuit, as shown in Fig. 4c [90]. Moreover, among the abundant variants of HNN, the Boltzmann machine (BM) was highlighted in the work due to the introduction of simulated annealing. The originally detrimental circuit noises were exploited and modulated by the applied voltage through the scaling circuit module, which in turn controlled the effective computing temperature of simulated annealing, as demonstrated in Fig. 4d. During the process of BM, the initially large applied voltage results in highly effective computing temperature, and the states of the neurons tend to flip randomly. As the network iterates, the applied voltage gradually decreases, and the network escapes from the local energy minimum and eventually gets a better convergence result.

Another inspiring example proposed by Dalgaty et al. achieved the Markov chain Monte Carlo (MCMC) sampling method utilizing the intrinsic memristor variability shown in Fig. 4e [91]. The MCMC methods optimize the problem solving by data sampling and can be further used for deep neural network optimization. The memristor crossbar array is used as a natural hardware sampling machine where each row of the array indicates a solution to the problem. The set programming currents of the (*n* + 1)th row of the array are controlled by the conductance of the *n*th row and applied to the gate voltages of the transistor in the three-terminal 1T1R structure. The newly produced conductance of the (*n* + 1)th array row satisfies the probability distribution where the mean value is consistent with the previous row and thus realizes the data sampling of the Metropolis–Hastings MCMC algorithm. The relationship of the set programming currents and the conductance is obtained by analyzing a large number of device properties. The hardware optimization machine is then used to achieve the supervised classification tasks and the reinforcement learning task. The obtained results are comparable with the software-based ones, and the required programming steps are between one and four orders of magnitude fewer than the memristive neural network benchmark models.

## Hard computing

Compared to soft computing that focuses on the associations of the data, hard computing highlights the specific numerical results of each task particularly, and is very sensitive to the noises during the computing [95]. Minor errors in intermediate results of the computation process will lead to large mistakes in the final results, just like the butterfly effect. Therefore, hard computing commonly requires higher computational accuracies, claiming demands on the effective solutions of at least one aspect of the severe challenges, such as the relatively large variations and other nonideal factors of the memristor devices, and the limited hardware computational precision. In this section, we introduce the recent progress in scientific computing and digital image processing as the representative hard computing tasks, and discuss the advanced strategies to address these remaining challenges and improve their computational accuracies in the IMC paradigm.

### Scientific computing

Scientific computing aims to model the natural and technological processes in scientific research and engineering, which is widely used in simulation, prediction, and optimization tasks such as weather forecast and economics. The construction of a high-performance scientific computing system should consider the computation precision, which is typically 32-bit floating point (FP) [96], the overall computation time complexity, and the computational efficiency. However, the memristive IMC, due to the limitation of the device non-ideal factors, can only perform low-precision computation (typically ≤ 6-bit). Besides that, although the memristive array can perform the *O*(1) time-complexity MVM, the overall IMC systems may not be able to carry out the solution for scientific computing with *O*(1) complexity due to the introduction of the peripheral circuit [97]. In addition, the computation efficiency of scientific computing problems will be significantly influenced by the applied strategy to perform sparse vector–matrix multiplication (SpVM) in the crossbar array. These issues all hinder the development of high-performance in-memory scientific computing systems. In this section, we will introduce several novel solutions of in-memory computing systems including the analog–digital co-processors and the pure-analog processors, which can overcome these disadvantages and address scientific computing, and further discuss the strategies to perform higher-efficient SpVM in a memristive array.

#### Analogue–digital co-processors

Modern high-precision computers are commonly relying on digital computation methods. Therefore, a straightforward method to overcome the low computation precision of the analog matrix multiplication is introducing the digital processing unit to form the analog–digital co-processors.

**Fig. 5:**
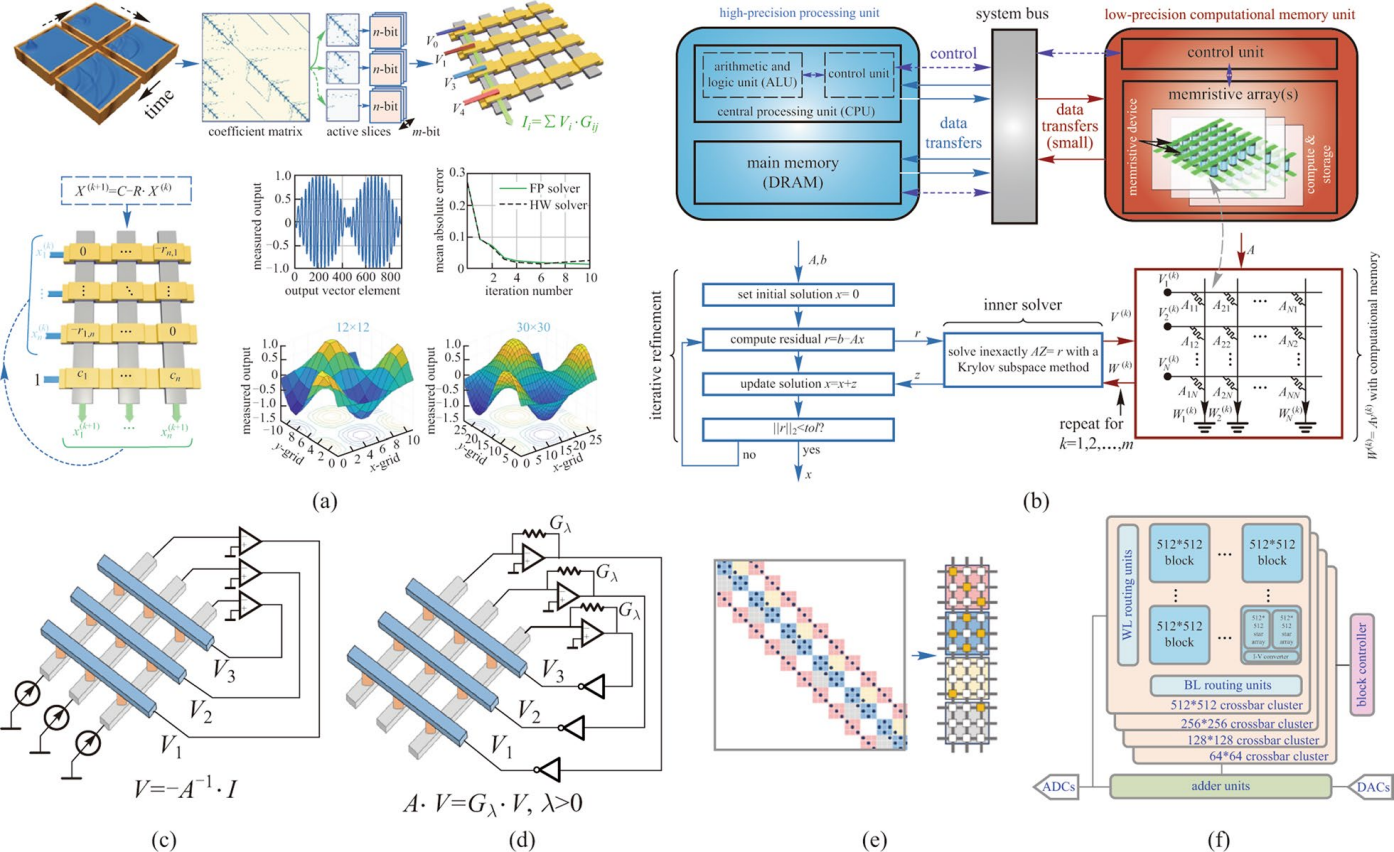
Concept of various scientific computing strategies. **a** Scheme of the bit-slice method, and solving PDEs in the bit-slice based analog–digital co-processors. Adapted from Ref. [48]. **b** Architecture of the mixed-precision ICM system, and the solution work-flow of solving matrix equation $Ax=b$ in the mixed-precision architecture. Adapted from Ref. [99]. **c** One-step matrix equation solving circuit. **d** One-step matrix eigenvector solving circuit. **c** and **d** are reproduced from Ref. [100]. **e** Matrix-slice method to perform SpVM. Adapted from Ref. [48]. **f** Scalable block mapping architecture. Adapted from Ref. [101]

From the array level, introducing the digital processing unit can extend the low natural computation precision of the memristor arrays. A prime method to implement the precision extension is performing the “bit-slice” method in the analog–digital cooperation MVM core. The concept of the bit-slice method is to support high-precision computation using the base-*n* operations, where *n* is the natural precision of the memristors [98]. Thus, multiple crossbar arrays are used to map the high-precision (*m*-bit) matrix, while each crossbar represents a portion (*n*-bit) of the desired precision. During computation, each crossbar array performs the *n*-bit precision analog matrix multiplication. And the partial product is extended to m bit by the digital shift-and-add units based on the binary arithmetic rules. Zidan et al. successfully demonstrated the bit-slice method by extending the computation precision of a 1-bit 16 × 3 Ta_2_O_5−*x*_ ReRAM array crossbar arrays to 16 bit [48]. This memristor-based hardware system was used to solve 2D static and time-evolving partial differential equations by performing the mathematical iteration method. The solution precision of the memristor hardware system is comparable with the FP digital computers (Fig. 5a). Such kind of analog–digital cooperation approach has also been used to improve the in-memory computation precision of other non-volatile memory such as the NAND/NOR flash [102].

From the system level, introducing the digital processor is capable of improving the overall computation precision of the memristor-based hardware system to solve the scientific computing problem. Le Gallo et al. proposed a mixed-precision IMC architecture combing the low-precision in-memory processing unit and the high-precision digital unit to solve the matrix equation [99], namely

2 $$Ax=b, A\in {R}^{N\times N}, b\in {R}^{N},$$

where *A* is the known non-singular matrix, *b* is the column vector, and *x* is the n-dimensional unknown vector to be solved. The mixed-precision solver utilized the Richardson refinement method [103] to solve the matrix equation with high-precision. In the hybrid system as demonstrated in Fig. 5b, a random *n*-dimensional vector is first initialized as the starting point, and then iteratively updated by the low-precision error-correction term *z*. The vector *z* is obtained by solving the matrix equation $Az=r$ in the inexact Krylov-solver with low-precision on the memristor array, where the residual vector *r* is provided by $r=b-Ax$ with high-precision on the digital computer. The solving algorithm iterates until the residual *r* reaches below the designed tolerance *ε*. This analog–digital co-processor can benefit from both the high computing efficiency of the IMC and the high computing precision of the digital processors. For experimental demonstration, a 5000-dimensional model covariance problem was mapped to a 1 Mb prototype PCM chip to validate the ability of the mixed-precision solver for real-world problems. To further improve the computing accuracy, the covariance matrix was mapped to 16 sub-arrays and averaged for redundancy to overcome the conductance drift of the PCM devices. The solution residual was reduced to 10^−6^ within 24 iteration cycles, approving the high-precision solving ability of the mixed-precision IMC architecture.

#### Pure-analog computing

Modern digital scientific computing processors commonly suffer from expensive computational time complexity as they rely on the iterative matrix multiplication methods to solve linear algebra problems. For instance, the typical solving time complexity of the CMOS processors is *O*(*N*^3^), where *N* is the size of the problem. Thus, the digital processors will be significantly hindered by computation latency in solving large-scale problems.

The pure-analog IMC paradigm serves as one of the most feasible solutions to significantly reduce the time complexity in solving linear algebra problems. Figure 5c shows the one-step matrix equation solving circuit proposed by Sun et al. [100], which was demonstrated using a 3 × 3 HfO_2_ ReRAM array and is able to accelerate the solution of matrix equation with *O*(1) time complexity. This approach is based on the idea of solving matrix inverse using the analog method. In this circuit, the principles of the memristors to perform the MAC operation can be written as $I=G\cdot V$. And the operational amplifiers (OAs) are added to the array to construct the TIAs, which were used to perform the inverse operation of $V={-G}^{-1}\cdot I$. To solve the matrix equation determined as $Ax=b$ in this circuit, matrix *A* is mapped as the conductance value of the devices and the vector *b* is mapped to the input current vector. The solution $x={A}^{-1}\cdot b$ will be provided by the voltage values within one step. Moreover, to overcome the disadvantage that the conductance of the devices can only map positive matrix elements, this circuit can be extended to solve the mixed-value (the matrix contains both positive and negative value) problems by connecting another crossbar array to the circuit to construct the analog difference structure. Further estimation indicates that the time complexity of the analog inverse solving circuit is approximate to *O*(1) [104].

Eigenvector calculation as an important linear-algebra problem can also be solved by the pure-analog circuit in one step. Based on the former research, Sun et al. further added another series of TIAs to the circuits with the feedback resistors ${G}_{\lambda }$ to solve the eigenvector equation determined as $Ax=\lambda x$, as demonstrated in Fig. 5d. The eigenvalue *λ* was mapped to the resistance value ${G}_{\lambda }$. Based on the circuit principles, the output of the eigenvector circuit could be written as $-A\cdot (V/{G}_{\lambda })=-V$, which satisfies the solution of the eigenvector equation. The computational time complexity of the eigenvector solving circuit is also estimated to be *O*(1) [105]. And this circuit has also shown the potential to accelerate the PageRank tasks [106].

Moreover, Sun et al. further extended such concept of solving matrix inverse using analog circuit to solve the Pseudo-inverse of the non-singular matrix [107]. Song et al. proposed hardware numerical iterative algorithms based on the thought of pure-analog computing. According to the simulation results, such analog-solver demonstrated strong scalability to solve large-scale problems with *O*(1) solution time complexity [101].

#### Sparse-vector matrix multiplication optimization

Traditionally, the in-memory MVM method needs to map the whole matrix into the crossbar array, which is efficient for dense matrix multiplication. However, most of the matrix multiplications performed in scientific computing are sparse. Since the zero elements are much more than the nonzero elements in the sparse matrices, performing the SpVM in crossbar arrays is not efficient nor accurate, as the zero-elements mapped to the low conductance values will accumulate sufficiently large deviations [108]. Thus, one of the major topics to optimize the performance of the in-memory scientific computing system is the design of the highly efficient SpVM strategy.

To take advantage of sparsity, several in-memory SpVM optimization methods have been proposed. Figure 5e shows the matrix-slice method proposed by Zidan et al. to perform the in-memory SpVM, where the crossbar arrays only map the sub-matrices with the nonzero elements [48]. This method has shown good potential to process giant sparse matrix in multiple small-scale memristive arrays while still providing high computing parallelism. Feng et al., from a different perspective, used the matrix compress techniques to optimize the in-memory SpVM [102]. In their works, the original sparse matrix will be compressed by the compress techniques like compress sparse row (CSR) or compress sparse column (CSC) operations, and then mapped into the crossbar array [109]. After that, the in-memory SpVM will be performed row-by-row or column-by-column in the crossbar array. Compared with the matrix slice method, the matrix compression method commonly has higher area efficiency but will sacrifice the computation parallelism. A scalable block sparse matrix mapping technique was also proposed by Song et al. for pure-analog computing [101]. The purpose of this technique is to partition the sparse matrix into properly sized sub-matrices so that all sub-matrices which contain nonzero elements can be mapped to the crossbar blocks according to the corresponding size, as shown in Fig. 5f. This technique relies on performing a search algorithm to find out all valid blocks that can cover all nonzero elements of a sparse matrix, and expand them recursively to the corresponding blocks.

Computation precision, solution time complexity, and SpVM optimization are the three key considerations to construct the in-memory scientific computing processors. Further comparison between the state-of-the-art hardware in-memory scientific computing processors, as shown in Table 2, indicates that the analog–digital coprocessors can typically provide higher processing precision from the system level. However, these coprocessors cannot significantly mitigate the solution time complexity. The pure-analog processors, on the contrary, have the lowest solution time complexity (nearly *O*(1)) and higher energy efficiency, but the analog computation precision is highly restricted by the device conductance resolution. Besides that, the overall performance of the in-memory scientific computing system can be significantly improved by the well-designed SpVM strategy.

**Table 2:** Comparison of the state-of-the-art in-memory scientific computing processors

Parameter	Analogue–digital coprocessors		Pure-analogue processor
	Mixed-precision solver [99]	Memristor-based pde solver [48]	One-step matrix equation solver [106]
Device	PCM	Memristor	Memristor
Conductance resolution (bit)	$\sim 7$	$1$	$6$
Array cost	16	16	1
System computation precision (bit)	FP-32	$16$	$6$
Precision improvement method	Redundancy array and iterative refinement	Bit-slice	–
SpVM optimization strategy	–	Matrix-slice	–
Time-complexity	$O({N}^{2})$	$O({N}^{2})$	$O(1)$
Performance /(TOPS·W^−1^)	2.43	60.1	342 (page-rank task)

### Digital image processing

**Fig. 6:**
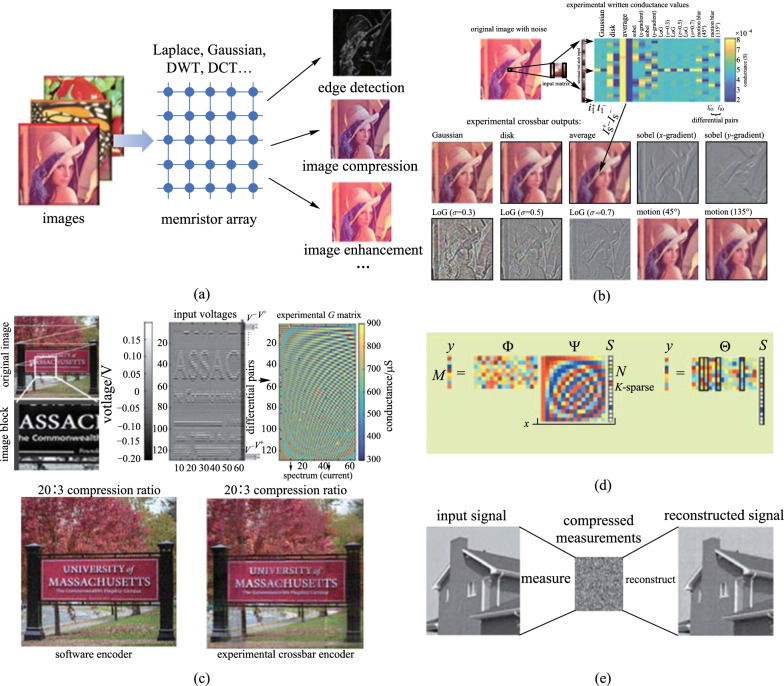
**a** Implementation of memristor-based image processing techniques. **b** Experimental convolution demonstration of edge detection and image filtering with differential memristor conductance pairs. **c** Experimental demonstration of image compression using 2D DCT. **a** and **b** are adapted from Ref. [49]. **d** Theory of compressive sensing. Adapted from Ref. [111]. **e** Illustration of compressive sensing measurement process. Adapted from Ref. [112].

Digital image processing is an important technology whose purposes are to improve the visual quality of images, extract the features for further image analysis, and perform data transformation and compression to facilitate image storage and communication [110]. With the development of computer science and mathematics, abundant approaches are proposed and take a key role in many fields such as aerospace, biomedicine, and robot vision. The essential mathematical computing procedures of many digital image processing algorithms involve a large number of MVM operations, such as convolution and orthogonal transformation. Therefore, owing to the abundant information of images, IMC demonstrates great potential in accelerating digital image processing (Fig. 6a). In this section, we take the image edge detection, compression, and compressive sense as examples to introduce how the IMC accelerates digital image processing in highly parallel, and foresee the future development trend of this field.

**Fig. 7:**
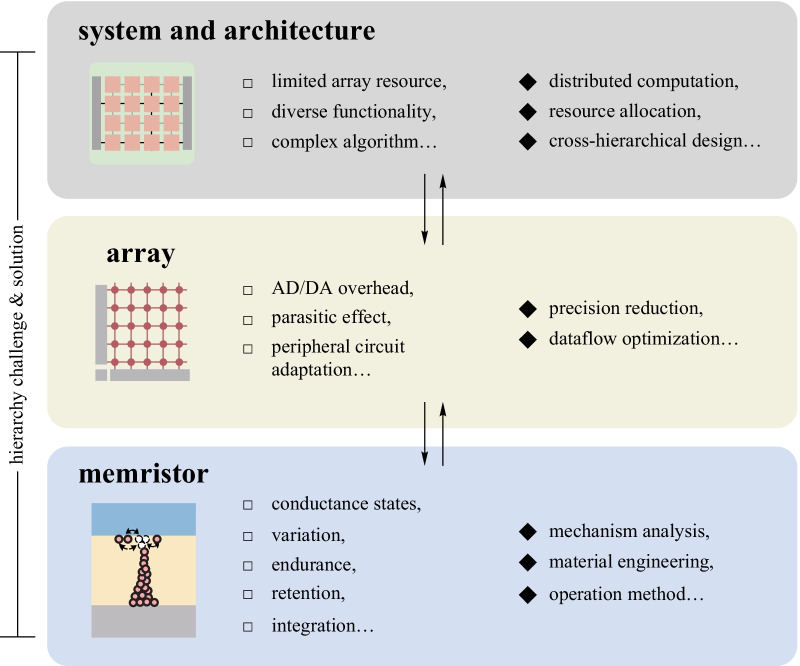
Hierarchical challenges and solutions for memristive in-memory computing

Edge detection is an image segmentation technique to minimize the processing data amount by preserving the important edge properties while filtering out useless data and noise of an image [113]. Basic image edge detection methods usually use convolutional operations to find the locations where the gray value changes abruptly, which are similar to those in the CNNs, except that the kernels are pre-defined rather than being trained. In 2018, Li et al. experimentally demonstrated the edge detection application using a fabricated large-scale 128 × 64 1T1R crossbar array, as illustrated in Fig. 6a [49]. The stable multilevel characteristic (6-bit) and high yield (> 99.8%) of the memristors make them capable of mapping various convolutional kernels with high accuracy. To improve the parallelism of the edge detection, ten different 5 × 5 convolutional kernels were mapped on the 25 × 20 subarray using the differential pairs to map both the positive and negative elements. The low latency, high energy efficiency, and low complexity of the memristor-based edge detection demonstrated in that work make it a promising path toward IoT applications. With the development of computer vision, 3D image processing becomes emerging especially for medical and video images. However, 3D image processing requires more computation and memory access than 2D image processing. Under these considerations, Huo et al. fabricated an 8-layers 3D memristor array to achieve the 3D convolution operations [114]. Self-rectifying memristors are used to fabricate the 3D array since the unique asymmetric IV characteristics allow them to suppress the sneak path current problem and achieve high-density integration without the help of the transistors. Compared to other traditional 3D crossbar arrays, the fabricated array structure introduced additional vertical partition and insulation across the layers, which enables better utilization of each unit and improves the overall process parallelism. 3D Prewitt kernels were successfully demonstrated on the array to extract the 2D edge surfaces of 3D handwritten digits. Furthermore, the low-conductance characteristic of the self-rectifying memristors is also beneficial to depress energy consumption.

Image compression technologies aim to reduce the storage space of image data or save the bandwidth of the transmission channel. Orthogonal transformation is one of the most important approaches to conduct image compression, such as discrete wavelet transform (DWT), discrete cosine transform (DCT), and so on. The essence of the DWT is two-step matrix multiplications which can be determined by

3 $$F=W\times I\times {W}^{\mathrm{T}},$$

4 $$f={W}^{\mathrm{T}}\times {F}^{\mathrm{^{\prime}}}\times W,$$

wh﻿ere $F$ and $F\mathrm{^{\prime}}$ is the original and the modulated frequency matrix respectively, $W$ is the transformation coefficient matrix, $I$ is the original input image, and $f$ is the decompressed image. Halawani et al. proposed a memristor-based image compression architecture that exploits a lossy 2D DWT [115]. In the forward transformation, $W$ was mapped as the memristor conductance values, and $I$ was converted into input voltages. After getting $F$, quantization and coding operations were performed in the digital domain to conduct compressing. In the reverse transformation, the operation for $W$ was the same, and $F^{\prime}$ was converted into input voltages. The proposed architecture is composed of a computational memristor crossbar, an intermediate memory array that stores the row-transformed coefficients, and a final memory that holds the compressed version of the image. Owing to the limited conductance level of memristor, 32 grayscale was used instead of the full 256 range. Meanwhile, as some of these coefficients are negative, mapping these values to a negative conductance is not possible. Instead, the polarity of the applied voltage pulse is complemented. Simulation results show that the proposed architecture provides significant improvements in energy efficiency, area, and performance compared to the CMOS implementation with a comparable complex wavelet-structural similarity index. Compared with DWT, DCT can achieve better image information compaction.

The working principle of DCT is similar to that of DWT except for the transformation coefficient matrix. Li et al. configured a 128 × 64 memristor array to implement the image compression using DCT as a typical example of the linear transformation [49]. As shown in Fig. 6c, in the forward transform transformation, the original image information was processed with two steps of matrix multiplication using the same memristor array to get the frequency matrix. As human eyes are not sensitive to low-frequency information, a similar visual effect can be obtained by only retaining the high-frequency information, which is mainly located in the upper left of the frequency matrix. In the work, the frequencies containing the top 15% of the spectral amplitudes were retained, equivalently achieving a compression ratio of 20:3. Next, in the reverse transformation, by inputting the frequency information and reversing the input and output of the array, the decompressed image was obtained. The energy efficiency of the system was over 119.7 TOPS/W with a readout time of 10 ns, which is expected to be further improved with larger vectors and matrices as well as more advanced circuit design. Based on Li et al.’s work, optimizations have been implemented by other researchers. In 2020, Zhang et al. further reduced the error of image compression and energy consumption by making improvements on the operation algorithm [116]. Traditional DCT needs to operate the MVM twice. If the result of the first MVM is influenced by the non-ideal variations of the devices, the error will be amplified in the second MVM. Under these considerations, they changed the two-step MVM into single-step MVM through linear transformation to reduce the number of MVM operations and analog deviation. Zhang et al. demonstrated the image compression on a 128 × 32 memristor array and proposed the array-level boosting method with spatial extended allocation to improve the compression accuracy [117]. The 128 × 32 memristor array was divided into ${N}_{\mathrm{s}}$ subarrays and used to operate the DCT jointly. The result was obtained by averaging the total results of the ${N}_{\mathrm{s}}$ subarrays. If the error of the MVM is conformed to the normal distribution, the variance of the average result can be reduced to 1/*N* of that of using a single memristor array to operate DCT. Thus, the image compression quality is improved. Nevertheless, the development of image compression using a memristive array is still greatly hampered by the variation and limited conductance states of the memristors and the precision of ADC/DAC, requiring further optimizations on devices and peripheral circuits.

Besides that, compressive sensing (CS) is another important technique in signal processing. The theory of CS pointed out that if the signal is sparse (compressible) in a certain orthogonal space, it can be sampled at a lower frequency (much lower than Nyquist sampling frequency [118]) and reconstructed accurately with high probability. The principles of CS are illustrated in Fig. 6d [111], where $y$ is the observed value, $x$ is the input signal, $\Phi$ is the measurement matrix, $S$ is the sparse coefficient, $\psi$ is the sparse matrix, and $\Theta$ is the sensing matrix. CS process consists of two parts: compressed sampling and reconstruction. The compressed sampling is achieved by solving $y=\Phi x$. The point of CS reconstruction is to restore $x$ by solving $y=\Theta S$ to get $S$ and solving $x=\psi S$ to get $x$. The implementations of CS using memristors have been researched intensively. Liu et al. proposed a new approach for robust compressed sampling using memristor devices, achieving *O*(*N*^2.5^) speed-up in overall complexity [119]. Qian et al. proposed a memristor-based compressive sensing circuit and developed an analytical model to evaluate the properties of analog random sensing matrices for compressive sensing applications [120]. The work further reduced the energy consumption and refined the compressed sampling study based on the memristor device. Yet, the main computational challenge of CS remains in reconstruction, which is an NP-Hard problem, and tends to be solved with norm (L1) minimization or other approximate estimation algorithms. Under these considerations, Le Gallo et al. proposed an experimental demonstration of both the compression sampling and reconstruction processes of CS with a 256 K-PCM shown in Fig. 6e [112]. Approximate message passing (AMP) algorithm was used in the work for reconstruction, and an *O*(*N*)-fold complexity reduction can be achieved compared with standard software-based approaches.

Traditional digital signal processing methods usually use analog signal sensors and digital processing units which are physically segmented from each other, leading to high latency and energy consumption for data transferring. IMC-based image processing has shown significant advantages not only in the parallel processing of MVM operations but also in taking advantage of the analog computing nature by reducing the distance between the signal sensing and processing in abundant techniques [121–124]. Furthermore, many new types of memristors, as well as other emerging nanodevices, have been developed as visual [125, 126], auditory [127], and olfactory [128] sensors, etc., generating excitation from the applied signals and processing them in situ, and greatly improving the compactness, processing speed, and energy efficiency. Many research efforts have been conducted toward constructing such systems that integrate sensing, storage, and computation together, which is termed in-sensor computing, paving the critical avenue of edge signal processing for the IoT applications [129–131].

## Conclusions and outlooks

In this review, we have discussed various trending application fields of memristive IMC with a comprehensive introduction of the algorithms and the development status. Soft computing applications as represented by machine learning have been extensively validated on memristive arrays in many sub-fields including neural networks, clustering, regression, and so on, with loose requirements on the memristor devices. Hard computing applications in memristor arrays have also obtained significant progress in reducing the energy consumption and time complexity in scientific computing and image processing. Regardless of the different algorithms and hardware requirements, such applications all greatly benefit from the high parallelism of executing MVM and eliminating a large amount of data movement. A recent study has even enabled the matrix–matrix multiplication (MMM) by utilizing a continuous-time data representation [132]. It can be expected that various applications that possess such MVM-intensive formats, especially for those with stationed and constant matrices during the execution, will obtain promising acceleration with memristive IMC. But there still exists considerable challenges in each level from the device characteristics to the system design, as illustrated in Fig. 7.

As for the memristor characteristics, the most critical performance metrics usually vary by application scenario. For applications where the matrices are transferred onto the arrays without frequent update requirements like neural network inference, scientific computing, and image processing, the accurate programming of the conductance and the retention is of vital importance, while the endurance is of less concern. But other applications that involve frequent and continuous updates of the matrixes, like neural network training, rely heavily on the efficient gradual programming and endurance of the devices. As for the variations that originate from intrinsic stochasticity, they are helpful for optimization problems and stochastic computing. Many machine learning tasks can tolerate moderate variations, while memristive neural network learning can compensate for a certain degree of device variations through the in situ training method. In contrast, hard computing tasks are sensitive to variations and usually require extra efforts to suppress the variations, especially for the scientific computing tasks. The lower conductance of the states, the lower operating voltages, and the forming-free characteristics are always preferable for most IMC applications to further suppress the IR drop and enhance the overall performance. Larger integration scale and density, even in 3D configuration, are also beneficial for more complex applications. Therefore, specific device optimizations should be conducted according to the different application domains through mechanism analysis, material engineering, and operation method optimization.

As for the array level, one major obstacle that hinders the performance of the memristive IMC is the large hardware overhead of the DACs and the ADCs for data in and out of the array. The higher precision of DACs and ADCs means more accurate analog computing results, but also brings larger hardware overhead including energy consumption, area, and latency, which will eventually lead to a complete loss of the advantages of IMC. For most soft computing tasks, the requirement of precision can be eased. Memristive neural network inference can generally be executed under low precision through the quantization algorithm. For other tasks that heavily rely on accurate MVM results, possible solutions are to use specially designed ADC/DAC arrays with optimized multiplexing and dataflow strategies [133]. Another promising solution is to use fixed analog periphery circuits to conduct pure-analog computing for simple tasks like MLPs or those tasks with self-iterations. Another important concern is the parasitic effects of the array, such as line resistance and parasitic capacitance. Different from the traditional program and read operations in an array for data storage, the ideal IMC paradigm turns on as many devices as possible for highly parallel MVM operations, leading to large currents flowing through the array. The line resistance will consume a considerable part of the voltage signals especially at the end of the signal lines, which is also known as the IR drop, resulting in unpredictable errors in the MVM results. The parasitic capacitance will affect the transmission of the voltage signals and limit the processing speed of MVM operation. The progress in technology node and integration density makes these issues more and more significant, placing an upper limit for the array size and the parallel operating amount. A thorough analysis of the array-level simulation should be conducted to assist the hardware design.

As for the system and architecture aspects, first of all, a prominent constraint is the mismatch between the large scale of the matrices and the limited size of today’s memristor arrays. For instance, a 50-layer ResNet-50 network possesses over 25 Mb weights [5], not to mention the practical commercial network models, while the state-of-the-art memristor macros are several Mb. Moreover, when considering a universal memristive neural network chip, one should be able to adopt the diverse interconnection structures of the neural networks, like the residual connections of the ResNets, the gate control operations of the LSTMs, and the various convolution operations with different granularities of the CNNs [54]. It is worth noting that besides the universal IMC chips, the dedicated ones, just like what ASIC is to CPU, should also become one of the development mainstreams in the future. These considerations all place high demands on the architecture design, including but not limited to the design of distributed computation and resource allocation. Secondly, for specific applications, memristive neural network learning demonstrates impressive potential in enhancing flexibility and capability, but drawbacks are located in the precision and the complex hardware mapping method. For scientific computing, it is anticipated that further improvement can be achieved in analog–digital co-processors with lower time-complexity and promised accuracy, and more hardware-friendly in-memory SpVM strategies can be proposed. Besides that, the combination of the analog–digital co-processor and the pure-analog processor to take advantage of both is also worth exploring and remains an open question. The aforementioned aspects all imply that the cross-level architecture design combing the hardware mapping and the software algorithm is necessary for more powerful and efficient memristive IMC systems.

Apart from IMC, neuromorphic computing is also an emerging approach to overcome the “von Neumann bottleneck” problem in traditional computing methods, which takes inspiration from the neuron system in the human brain [134–137]. Many people may confuse these two concepts. But as one can see from the presentation of our Review, these two are not identical. In IMC, memristors are used to map the values of the processed matrices. But in neuromorphic computing, memristors aim to imitate the various dynamic biological behaviors of the neurons. In neuromorphic computing, a most typical approach is to use the analog conductance tuning behavior of the memristors to simulate the long-term potentiation/depression (LTP/LTD) behavior of biological synapses, which in turn can be used for many ANN applications, as been described in Sect. 3. This may to some extent explain why these two concepts are easily confused. But memristive neuromorphic computing is much more than that. Memristors can be used to imitate spike-timing-dependent plasticity (STDP) behavior, since that the conductance tuning behavior depends on the timing and interval of the applied pre- and post-pulses. Such characteristics can be utilized as the basic learning rules for spike neural networks (SNNs) [138, 139]. Many generalized memristors with volatile properties, which are also referred to as diffusive memristors, can be used to implement the leaky integrate-and-fire (LIF) neuron model [140, 141]. Memristive artificial dendrites have also been investigated to achieve more complex and bio-fidelic artificial neurons with higher signal processing capability, and construct a more complete bio-inspired neural network [142]. Recent advanced researches further concentrated on implementing brain-like dynamic computing by integrating dynamic characteristics of synapses, dendrites, neurons, and bio-inspired somatosensory systems realized by neuromorphic memristors [143–145]. In general, it is clear that these two concepts are different from each other, but IMC can serve as a promising approach for implementing neuromorphic computing.

In conclusion, memristive IMC has demonstrated significant progress in processing speed and energy efficiency compared to traditional computing systems. We believe that memristors and the IMC paradigm will continue to progress in leaps and bounds with the efforts from the device to the architecture levels, and obtain immense accomplishment in the fields of IoT and edge intelligence.

## References

[1] Jordan MI, Mitchell TM (2015). Machine learning: trends, perspectives, and prospects. Science.

[2] Kuznetsova A, Rom H, Alldrin N, Uijlings J, Krasin I, Pont-Tuset J, Kamali S, Popov S, Malloci M, Kolesnikov A, Duerig T, Ferrari V (2020). The open images dataset v4. Int. J. Comput. Vis..

[3] Deng, J., Dong, W., Socher, R., Li, L.J., Li, K., Fei-Fei, L.: Imagenet: a large-scale hierarchical image database. In: Proceedings of 2009 IEEE Conference on Computer Vision and Pattern Recognition. IEEE, 248–255 (2009)

[4] Simonyan, K., Zisserman, A. Very deep convolutional networks for large-scale image recognition. arXiv preprint arXiv:14091556 (2014)

[5] He, K., Zhang, X., Ren, S., Sun, J. Deep residual learning for image recognition. In: Proceedings of the IEEE Conference on Computer Vision and Pattern Recognition. IEEE, 770–778 (2016)

[6] Keckler SW, Dally WJ, Khailany B, Garland M, Glasco D (2011). GPUs and the future of parallel computing. IEEE Micro.

[7] Owens JD, Houston M, Luebke D, Green S, Stone JE, Phillips JC (2008). GPU computing. Proc. IEEE.

[8] Mutlu O, Ghose S, Gómez-Luna J, Ausavarungnirun R (2019). Processing data where it makes sense: enabling in-memory computation. Microprocess. Microsyst..

[9] Chua LO (2018). How we predicted the memristor. Nat. Electron..

[10] Williams RS, Tetzlaff R (2014). How we found the missing memristor. Memristors and memristive systems.

[11] Chua L (1971). Memristor—the missing circuit element. IEEE Trans Circuit Theory.

[12] Strukov DB, Snider GS, Stewart DR, Williams RS (2008). The missing memristor found. Nature.

[13] Zidan MA, Strachan JP, Lu WD (2018). The future of electronics based on memristive systems. Nat. Electron..

[14] Lee J, Lu WD (2018). On-demand reconfiguration of nanomaterials: when electronics meets ionics. Adv. Mater..

[15] Sun W, Gao B, Chi M, Xia Q, Yang JJ, Qian H, Wu H (2019). Understanding memristive switching via in situ characterization and device modeling. Nat. Commun..

[16] Cheng L, Li Y, Yin KS, Hu SY, Su YT, Jin MM, Wang ZR, Chang TC, Miao XS (2019). Functional demonstration of a memristive arithmetic logic unit (MemALU) for in-memory computing. Adv. Func. Mater..

[17] Yang L, Cheng L, Li Y, Li H, Li J, Chang TC, Miao X (2021). Cryptographic key generation and in situ encryption in one-transistor-one-resistor memristors for hardware security. Adv. Electron. Mater..

[18] Karunaratne G, Le Gallo M, Cherubini G, Benini L, Rahimi A, Sebastian A (2020). In-memory hyperdimensional computing. Nat. Electron..

[19] Junsangsri, P., Lombardi, F.: A memristor-based TCAM (ternary content addressable memory) cell: design and evaluation. In: Proceedings of the Great Lakes Symposium on VLSI. ACM, 311–314 (2012)

[20] Graves CE, Li C, Sheng X, Miller D, Ignowski J, Kiyama L, Strachan JP (2020). In-memory computing with memristor content addressable memories for pattern matching. Adv. Mater..

[21] Hu M, Graves CE, Li C, Li Y, Ge N, Montgomery E, Davila N, Jiang H, Williams RS, Yang JJ, Xia Q, Strachan JP (2018). Memristor-based analog computation and neural network classification with a dot product engine. Adv. Mater..

[22] Yao P, Wu H, Gao B, Eryilmaz SB, Huang X, Zhang W, Zhang Q, Deng N, Shi L, Wong HP, Qian H (2017). Face classification using electronic synapses. Nat. Commun..

[23] Amirsoleimani A, Alibart F, Yon V, Xu J, Pazhouhandeh MR, Ecoffey S, Beilliard Y, Genov R, Drouin D (2020). In-memory vector-matrix multiplication in monolithic complementary metal–oxide–semiconductor-memristor integrated circuits: design choices, challenges, and perspectives. Adv. Intell. Syst..

[24] Xia Q, Yang JJ (2019). Memristive crossbar arrays for brain-inspired computing. Nat. Mater..

[25] Yan B, Li B, Qiao X, Xue CX, Chang MF, Chen Y, Li H (2019). Resistive memory-based in-memory computing: from device and large-scale integration system perspectives. Adv. Intell. Syst..

[26] Zhang T, Yang K, Xu X, Cai Y, Yang Y, Huang R (2019). Memristive devices and networks for brain-inspired computing. Phys. Status Solidi (RRL) Rapid Res. Lett..

[27] Shi T, Wang R, Wu Z, Sun Y, An J, Liu Q (2021). A review of resistive switching devices: performance improvement, characterization, and applications. Small Struct..

[28] Hung JM, Jhang CJ, Wu PC, Chiu YC, Chang MF (2020). Challenges and trends of nonvolatile in-memory-computation circuits for AI edge devices. IEEE Trans. Electron Devices.

[29] Guo, X., Bayat, F.M., Bavandpour, M., Klachko, M., Mahmoodi, M., Prezioso, M., Likharev, K., Strukov D.: Fast, energy-efficient, robust, and reproducible mixed-signal neuromorphic classifier based on embedded NOR flash memory technology. In: Proceedings of 2017 IEEE International Electron Devices Meeting (IEDM). IEEE, 6.5.1–6.5.4 (2017)

[30] Ambrogio S, Narayanan P, Tsai H, Shelby RM, Boybat I, di Nolfo C, Sidler S, Giordano M, Bodini M, Farinha NCP, Killeen B, Cheng C, Jaoudi Y, Burr GW (2018). Equivalent-accuracy accelerated neural-network training using analogue memory. Nature.

[31] Ni K, Yin X, Laguna AF, Joshi S, Duenkel S, Trentzsch M, Müller J, Beyer S, Niemier M, Hu XS (2019). Ferroelectric ternary content-addressable memory for one-shot learning. Nat. Electron..

[32] Jung S, Lee H, Myung S, Kim H, Yoon SK, Kwon SW, Ju Y, Kim M, Yi W, Han S, Kwon B, Seo B, Lee K, Koh GH, Lee K, Song Y, Choi C, Ham D, Kim SJ (2022). A crossbar array of magnetoresistive memory devices for in-memory computing. Nature.

[33] Chen J, Li J, Li Y, Miao X (2021). Multiply accumulate operations in memristor crossbar arrays for analog computing. J. Semicond..

[34] Sebastian A, Le Gallo M, Khaddam-Aljameh R, Eleftheriou E (2020). Memory devices and applications for in-memory computing. Nat. Nanotechnol..

[35] Qin YF, Bao H, Wang F, Chen J, Li Y, Miao XS (2020). Recent progress on memristive convolutional neural networks for edge intelligence. Adv. Intell. Syst..

[36] Ibrahim D (2016). An overview of soft computing. Procedia Comput. Sci..

[37] Yin S, Sun X, Yu S, Seo J (2020). High-throughput in-memory computing for binary deep neural networks with monolithically integrated RRAM and 90-nm CMOS. IEEE Trans. Electron Devices.

[38] Yu, S., Li, Z., Chen, P.Y., Wu, H., Gao, B., Wang, D., Wu, W., Qian H.: Binary neural network with 16 Mb RRAM macro chip for classification and online training. In: Proceedings of 2016 IEEE International Electron Devices Meeting (IEDM). IEEE, 16.2.1–16.2.4 (2016)

[39] Xue CX, Chiu YC, Liu TW, Huang TY, Liu JS, Chang TW, Kao HY, Wang JH, Wei SY, Lee CY, Huang SP, Hung JM, Teng SH, Wei WC, Chen YR, Hsu TH, Chen YK, Lo YC, Wen TH, Lo CC, Liu RS, Hsieh CC, Tang KT, Ho MS, Su CY, Chou CC, Chih YD, Chang MF (2021). A CMOS-integrated compute-in-memory macro based on resistive random-access memory for AI edge devices. Nat. Electron..

[40] Kim H, Mahmoodi MR, Nili H, Strukov DB (2021). 4K-memristor analog-grade passive crossbar circuit. Nat. Commun..

[41] Yao P, Wu H, Gao B, Tang J, Zhang Q, Zhang W, Yang JJ, Qian H (2020). Fully hardware-implemented memristor convolutional neural network. Nature.

[42] Wang Z, Li C, Lin P, Rao M, Nie Y, Song W, Qiu Q, Li Y, Yan P, Strachan JP, Ge N, McDonald N, Wu Q, Hu M, Wu H, Williams RS, Xia Q, Yang JJ (2019). In situ training of feed-forward and recurrent convolutional memristor networks. Nat. Mach. Intell..

[43] Wang Z, Li C, Song W, Rao M, Belkin D, Li Y, Yan P, Jiang H, Lin P, Hu M, Strachan JP, Ge N, Barnell M, Wu Q, Barto AG, Qiu Q, Williams RS, Xia Q, Yang JJ (2019). Reinforcement learning with analogue memristor arrays. Nat. Electron..

[44] Li C, Wang Z, Rao M, Belkin D, Song W, Jiang H, Yan P, Li Y, Lin P, Hu M, Ge N, Strachan JP, Barnell M, Wu Q, Williams RS, Yang JJ, Xia Q (2019). Long short-term memory networks in memristor crossbar arrays. Nat. Mach. Intell..

[45] Li C, Belkin D, Li Y, Yan P, Hu M, Ge N, Jiang H, Montgomery E, Lin P, Wang Z, Song W, Strachan JP, Barnell M, Wu Q, Williams RS, Yang JJ, Xia Q (2018). Efficient and self-adaptive *in-situ* learning in multilayer memristor neural networks. Nat. Commun..

[46] Cai F, Correll JM, Lee SH, Lim Y, Bothra V, Zhang Z, Flynn MP, Lu WD (2019). A fully integrated reprogrammable memristor–CMOS system for efficient multiply–accumulate operations. Nat. Electron..

[47] Sheridan PM, Cai F, Du C, Ma W, Zhang Z, Lu WD (2017). Sparse coding with memristor networks. Nat. Nanotechnol..

[48] Zidan MA, Jeong Y, Lee J, Chen B, Huang S, Kushner MJ, Lu WD (2018). A general memristor-based partial differential equation solver. Nat. Electron..

[49] Li C, Hu M, Li Y, Jiang H, Ge N, Montgomery E, Zhang J, Song W, Dávila N, Graves CE, Li Z, Strachan JP, Lin P, Wang Z, Barnell M, Wu Q, Williams RS, Yang JJ, Xia Q (2018). Analogue signal and image processing with large memristor crossbars. Nat. Electron..

[50] LeCun Y, Bengio Y, Hinton G (2015). Deep learning. Nature.

[51] Sainath TN, Kingsbury B, Saon G, Soltau H, Mohamed AR, Dahl G, Ramabhadran B (2015). Deep convolutional neural networks for large-scale speech tasks. Neural Netw..

[52] Krizhevsky A, Sutskever I, Hinton GE (2012). Imagenet classification with deep convolutional neural networks. Adv. Neural. Inf. Process. Syst..

[53] Girshick, R., Donahue, J., Darrell, T., Malik, J.: Rich feature hierarchies for accurate object detection and semantic segmentation. In: Proceedings of the IEEE Conference on Computer Vision and Pattern Recognition. IEEE, 580–587 (2014)

[54] Sze V, Chen YH, Yang TJ, Emer JS (2017). Efficient processing of deep neural networks: a tutorial and survey. Proc. IEEE.

[55] Bayat FM, Prezioso M, Chakrabarti B, Nili H, Kataeva I, Strukov D (2018). Implementation of multilayer perceptron network with highly uniform passive memristive crossbar circuits. Nat. Commun..

[56] Lin P, Li C, Wang Z, Li Y, Jiang H, Song W, Rao M, Zhuo Y, Upadhyay NK, Barnell M, Wu Q, Yang JJ, Xia Q (2020). Three-dimensional memristor circuits as complex neural networks. Nat. Electron..

[57] Li T, Yin Y, Ma K, Zhang S, Liu M (2021). Lightweight end-to-end neural network model for automatic heart sound classification. Information (Basel).

[58] Karunaratne G, Schmuck M, Le Gallo M, Cherubini G, Benini L, Sebastian A, Rahimi A (2021). Robust high-dimensional memory-augmented neural networks. Nat. Commun..

[59] Li, H., Chen, W.C., Levy, A., Wang, C.H., Wang, H., Chen, P.H., Wan, W., Wong, H.S.P., Raina, P.: One-shot learning with memory-augmented neural networks using a 64-kbit, 118 GOPS/W RRAM-based non-volatile associative memory. In: Proceedings of 2021 Symposium on VLSI Technology. IEEE, 1–2 (2021)

[60] Wu, S., Li, G., Chen, F., Shi, L.: Training and inference with integers in deep neural networks. arXiv preprint arXiv:180204680 (2018)

[61] Zhang Q, Wu H, Yao P, Zhang W, Gao B, Deng N, Qian H (2018). Sign backpropagation: an on-chip learning algorithm for analog RRAM neuromorphic computing systems. Neural Netw..

[62] Gokmen T, Onen M, Haensch W (2017). Training deep convolutional neural networks with resistive cross-point devices. Front. Neurosci..

[63] Lim S, Bae JH, Eum JH, Lee S, Kim CH, Kwon D, Park BG, Lee JH (2019). Adaptive learning rule for hardware-based deep neural networks using electronic synapse devices. Neural Comput. Appl..

[64] Geng, Y., Gao, B., Zhang, Q., Zhang, W., Yao, P., Xi, Y., Lin, Y., Chen, J., Tang, J., Wu, H.: An on-chip layer-wise training method for RRAM based computing-in-memory chips. In: Proceedings of 2021 Design, Automation and Test in Europe Conference and Exhibition (DATE). IEEE, 248–251 (2021)

[65] Jiang, H., Huang, S., Peng, X., Yu, S.: MINT: Mixed-precision RRAM-based IN-memory training architecture. In: Proceedings of 2020 IEEE International Symposium on Circuits and Systems (ISCAS). IEEE, 1–5 (2020)

[66] Negrov D, Karandashev I, Shakirov V, Matveyev Y, Dunin-Barkowski W, Zenkevich A (2017). An approximate backpropagation learning rule for memristor based neural networks using synaptic plasticity. Neurocomputing.

[67] Lillicrap TP, Cownden D, Tweed DB, Akerman CJ (2016). Random synaptic feedback weights support error backpropagation for deep learning. Nat. Commun..

[68] Lu, Y., Li, X., Yan, L., Zhang, T., Yang, Y., Song, Z., Huang R.: Accelerated local training of CNNs by optimized direct feedback alignment based on stochasticity of 4 Mb C-doped Ge_2_Sb_2_Te_5_ PCM chip in 40 nm node. In: Proceedings of 2020 IEEE International Electron Devices Meeting (IEDM). IEEE, 36.33.31–36.33.34 (2020)

[69] Luo Y, Han X, Ye Z, Barnaby H, Seo JS, Yu S (2020). Array-level programming of 3-bit per cell resistive memory and its application for deep neural network inference. IEEE Trans. Electron Devices.

[70] Chen J, Pan WQ, Li Y, Kuang R, He YH, Lin CY, Duan N, Feng GR, Zheng HX, Chang TC, Sze SM, Miao XS (2020). High-precision symmetric weight update of memristor by gate voltage ramping method for convolutional neural network accelerator. IEEE Electron Device Lett..

[71] Cai Y, Tang T, Xia L, Li B, Wang Y, Yang H (2020). Low bit-width convolutional neural network on RRAM. IEEE Trans. Comput. Aided Des. Integr. Circuits Syst..

[72] Hubara I, Courbariaux M, Soudry D, El-Yaniv R, Bengio Y (2017). Quantized neural networks: training neural networks with low precision weights and activations. J. Mach. Learn. Res..

[73] Qin YF, Kuang R, Huang XD, Li Y, Chen J, Miao XS (2020). Design of high robustness BNN inference accelerator based on binary memristors. IEEE Trans. Electron Devices.

[74] Pan WQ, Chen J, Kuang R, Li Y, He YH, Feng GR, Duan N, Chang TC, Miao XS (2020). Strategies to improve the accuracy of memristor-based convolutional neural networks. IEEE Trans. Electron Devices.

[75] Xi Y, Gao B, Tang J, Chen A, Chang MF, Hu XS, Spiegel JVD, Qian H, Wu H (2021). In-memory learning with analog resistive switching memory: a review and perspective. Proc. IEEE.

[76] Kim SG, Han JS, Kim H, Kim SY, Jang HW (2018). Recent advances in memristive materials for artificial synapses. Adv. Mater. Technol..

[77] Chen J, Lin CY, Li Y, Qin C, Lu K, Wang JM, Chen CK, He YH, Chang TC, Sze SM, Miao XS (2019). LiSiO X-based analog memristive synapse for neuromorphic computing. IEEE Electron Device Lett..

[78] Yu, S:. Orientation classification by a winner-take-all network with oxide RRAM based synaptic devices. In: Proceedings of 2014 IEEE International Symposium on Circuits and Systems (ISCAS). IEEE, 1058–1061 (2014)

[79] Jiang Y, Kang J, Wang X (2017). RRAM-based parallel computing architecture using k-nearest neighbor classification for pattern recognition. Sci. Rep..

[80] Jeong Y, Lee J, Moon J, Shin JH, Lu WD (2018). K-means data clustering with memristor networks. Nano Lett..

[81] Zhou H, Chen J, Wang Y, Liu S, Li Y, Li Q, Liu Q, Wang Z, He Y, Xu H (2021). Energy-efficient memristive Euclidean distance engine for brain-inspired competitive learning. Adv. Intell. Syst..

[82] Choi S, Shin JH, Lee J, Sheridan P, Lu WD (2017). Experimental demonstration of feature extraction and dimensionality reduction using memristor networks. Nano Lett..

[83] Zhou H, Li Y, Miao X (2022). Low-time-complexity document clustering using memristive dot product engine. Science China. Inf. Sci..

[84] Milo, V., Anzalone, F., Zambelli, C., Pérez, E., Mahadevaiah, M.K., Ossorio, Ó.G., Olivo, P., Wenger, C., Ielmini, D.: Optimized programming algorithms for multilevel RRAM in hardware neural networks. In: Proceedings of 2021 IEEE International Reliability Physics Symposium (IRPS). IEEE, 1–6 (2021)

[85] Wang Z, Joshi S, Savel’ev SE, Jiang H, Midya R, Lin P, Hu M, Ge N, Strachan JP, Li Z, Wu Q, Barnell M, Li GL, Xin HL, Williams RS, Xia Q, Yang JJ (2017). Memristors with diffusive dynamics as synaptic emulators for neuromorphic computing. Nat. Mater..

[86] Chen, P.Y., Peng, X., Yu, S.: NeuroSim+: an integrated device-to-algorithm framework for benchmarking synaptic devices and array architectures. In: Proceedings of 2017 IEEE International Electron Devices Meeting (IEDM). IEEE, 6.1.1–6.1.4 (2017)

[87] Hopfield JJ (1982). Neural networks and physical systems with emergent collective computational abilities. Proc. Natl. Acad. Sci. U.S.A..

[88] Cai F, Kumar S, Van Vaerenbergh T, Sheng X, Liu R, Li C, Liu Z, Foltin M, Yu S, Xia Q, Yang JJ, Beausoleil R, Lu WD, Strachan JP (2020). Power-efficient combinatorial optimization using intrinsic noise in memristor Hopfield neural networks. Nat. Electron..

[89] Yang K, Duan Q, Wang Y, Zhang T, Yang Y, Huang R (2020). Transiently chaotic simulated annealing based on intrinsic nonlinearity of memristors for efficient solution of optimization problems. Sci Adv.

[90] Mahmoodi MR, Prezioso M, Strukov DB (2019). Versatile stochastic dot product circuits based on nonvolatile memories for high performance neurocomputing and neurooptimization. Nat. Commun..

[91] Dalgaty T, Castellani N, Turck C, Harabi KE, Querlioz D, Vianello E (2021). In situ learning using intrinsic memristor variability via Markov chain Monte Carlo sampling. Nat. Electron..

[92] Chen L, Aihara K (1995). Chaotic simulated annealing by a neural network model with transient chaos. Neural Netw..

[93] Lu J, Wu Z, Zhang X, Wei J, Fang Y, Shi T, Liu Q, Wu F, Liu M (2020). Quantitatively evaluating the effect of read noise in memristive Hopfield network on solving traveling salesman problem. IEEE Electron Device Lett..

[94] Fahimi Z, Mahmoodi MR, Nili H, Polishchuk V, Strukov DB (2021). Combinatorial optimization by weight annealing in memristive hopfield networks. Sci. Rep..

[95] Ovaska SJ, VanLandingham HF, Kamiya A (2002). Fusion of soft computing and hard computing in industrial applications: an overview. IEEE Trans. Syst. Man Cybern. Part C Appl. Rev..

[96] Baboulin M, Buttari A, Dongarra J, Kurzak J, Langou J, Langou J, Luszczek P, Tomov S (2009). Accelerating scientific computations with mixed precision algorithms. Comput. Phys. Commun..

[97] Sun Z, Huang R (2021). Time complexity of in memory matrix vector multiplication. IEEE Trans. Circuits Syst. II Express Briefs.

[98] Feinberg, B., Vengalam, U.K.R., Whitehair, N., Wang, S., Ipek, E.: Enabling scientific computing on memristive accelerators. In: Proceedings of 2018 ACM/IEEE 45th Annual International Symposium on Computer Architecture (ISCA). IEEE, 367–382 (2018)

[99] Le Gallo M, Sebastian A, Mathis R, Manica M, Giefers H, Tuma T, Bekas C, Curioni A, Eleftheriou E (2018). Mixed-precision in-memory computing. Nat. Electron..

[100] Sun Z, Pedretti G, Ambrosi E, Bricalli A, Wang W, Ielmini D (2019). Solving matrix equations in one step with cross-point resistive arrays. Proc. Natl. Acad. Sci. U.S.A..

[101] Song, T., Chen, X., Han, Y.: Eliminating iterations of iterative methods: solving large-scale sparse linear system in *O*(1) with RRAM-based in-memory accelerator. In: Proceedings of the 2021 on Great Lakes Symposium on VLSI. ACM, 71–76 (2021)

[102] Feng, Y., Zhan, X., Chen, J.: Flash memory based computing-in-memory to solve time-dependent partial differential equations. In: Proceedings of 2020 IEEE Silicon Nanoelectronics Workshop (SNW). IEEE, 27–28 (2020)

[103] Kalantzis, V., Gupta, A., Horesh, L., Nowicki, T., Squillante, M. S., Wu, C. W., Gokmen, T., Avron, H.: Solving sparse linear systems with approximate inverse preconditioners on analog devices. arXiv preprint arXiv:210706973 (2021)

[104] Sun Z, Pedretti G, Mannocci P, Ambrosi E, Bricalli A, Ielmini D (2020). Time complexity of in-memory solution of linear systems. IEEE Trans. Electron Devices.

[105] Sun Z, Pedretti G, Ambrosi E, Bricalli A, Ielmini D (2020). In-memory eigenvector computation in time *O*(1). Adv. Intell. Syst..

[106] Sun Z, Ambrosi E, Pedretti G, Bricalli A, Ielmini D (2020). In-memory PageRank accelerator with a cross-point array of resistive memories. IEEE Trans. Electron Devices.

[107] Sun Z, Pedretti G, Bricalli A, Ielmini D (2020). One-step regression and classification with cross-point resistive memory arrays. Sci. Adv..

[108] Buluc, A., Gilbert, J. R.: Challenges and advances in parallel sparse matrix-matrix multiplication. In: Proceedings of 2008 37th International Conference on Parallel Processing. IEEE, 503–510 (2008)

[109] Borštnik U, VandeVondele J, Weber V, Hutter J (2014). Sparse matrix multiplication: the distributed block-compressed sparse row library. Parallel Comput..

[110] Pitas I (2000). Digital image processing algorithms and applications.

[111] Baraniuk RG (2007). Compressive sensing. IEEE Signal Process. Mag..

[112] Le Gallo, M., Sebastian, A., Cherubini, G., Giefers, H., Eleftheriou, E.: Compressed sensing recovery using computational memory. In: Proceedings of 2017 IEEE International Electron Devices Meeting (IEDM). IEEE, 28.23.21–28.23.24 (2017)

[113] Canny J (1986). A computational approach to edge detection. IEEE Trans. Pattern Anal. Mach. Intell..

[114] Huo Q, Song R, Lei D, Luo Q, Wu Z, Wu Z, Zhao X, Zhang F, Li L, Liu M (2020). Demonstration of 3D convolution kernel function based on 8-layer 3D vertical resistive random access memory. IEEE Electron Device Lett..

[115] Halawani Y, Mohammad B, Al-Qutayri M, Al-Sarawi SF (2018). Memristor-based hardware accelerator for image compression. IEEE Trans. VLSI Syst..

[116] Zhang, B., Uysal, N., Ewetz, R.: Computational restructuring: rethinking image processing using memristor crossbar arrays. In: Proceedings of 2020 Design, Automation and Test in Europe Conference and Exhibition (DATE). IEEE, 1594–1597 (2020)

[117] Zhang W, Gao B, Yao P, Tang J, Qian H, Wu H (2021). Array-level boosting method with spatial extended allocation to improve the accuracy of memristor based computing-in-memory chips. Science China. Inf. Sci..

[118] Oppenheim AV, Schafer RW, Buck JR (1999). Discrete-time signal processing.

[119] Liu, S., Ren, A., Wang, Y., Varshney, P. K.: Ultra-fast robust compressive sensing based on memristor crossbars. In: Proceedings of 2017 IEEE International Conference on Acoustics, Speech and Signal Processing (ICASSP). IEEE, 1133–1137 (2017)

[120] Qian, F., Gong, Y., Huang, G., Ahi, K., Anwar, M., Wang, L.: A memristor-based compressive sensing architecture. In: Proceedings of 2016 IEEE/ACM International Symposium on Nanoscale Architectures (NANOARCH). IEEE, 109–114 (2016)

[121] Zhao H, Liu Z, Tang J, Gao B, Zhang Y, Qian H, Wu H (2022). Memristor-based signal processing for edge computing. Tsinghua Sci. Technol..

[122] Zhu R, Tang Z, Ye S, Huang Q, Guo L, Chang S (2021). Memristor-based image enhancement: high efficiency and robustness. IEEE Trans. Electron Devices.

[123] Ran H, Wen S, Wang S, Cao Y, Zhou P, Huang T (2021). Memristor-based edge computing of ShuffleNetV2 for image classification. IEEE Trans. Comput. Aided Des. Integr. Circuits Syst..

[124] Hong Q, Li Y, Wang X (2020). Memristive continuous Hopfield neural network circuit for image restoration. Neural Comput. Appl..

[125] Mennel L, Symonowicz J, Wachter S, Polyushkin DK, Molina-Mendoza AJ, Mueller T (2020). Ultrafast machine vision with 2D material neural network image sensors. Nature.

[126] Zhou F, Zhou Z, Chen J, Choy TH, Wang J, Zhang N, Lin Z, Yu S, Kang J, Wong HP, Chai Y (2019). Optoelectronic resistive random access memory for neuromorphic vision sensors. Nat. Nanotechnol..

[127] Sun L, Zhang Y, Hwang G, Jiang J, Kim D, Eshete YA, Zhao R, Yang H (2018). Synaptic computation enabled by joule heating of single-layered semiconductors for sound localization. Nano Lett..

[128] Iwata, T., Ono, K., Yoshikawa, T., Sawada, K.: Gas discrimination based on single-device extraction of transient sensor response by a MetalOxide memristor toward olfactory sensor array. In: Proceedings of 2019 IEEE Sensors. IEEE, 1–4 (2019)

[129] Zhou F, Chai Y (2020). Near-sensor and in-sensor computing. Nat. Electron..

[130] Chai Y (2020). In-sensor computing for machine vision. Nature.

[131] Tong L, Peng Z, Lin R, Li Z, Wang Y, Huang X, Xue KH, Xu H, Liu F, Xia H, Wang P, Xu M, Xiong W, Hu W, Xu J, Zhang X, Ye L, Miao X (2021). 2D materials-based homogeneous transistor-memory architecture for neuromorphic hardware. Science.

[132] Wang C, Liang SJ, Wang CY, Yang ZZ, Ge Y, Pan C, Shen X, Wei W, Zhao Y, Zhang Z, Cheng B, Zhang C, Miao F (2021). Scalable massively parallel computing using continuous-time data representation in nanoscale crossbar array. Nat. Nanotechnol..

[133] Ankit, A., Hajj, I.E., Chalamalasetti, S.R., Ndu, G., Foltin, M., Williams, R. S., Faraboschi, P., Hwu, W. W., Strachan, J.P., Roy, K.: PUMA: a programmable ultra-efficient memristor-based accelerator for machine learning inference. In: Proceedings of 24th International Conference on Architectural Support for Programming Languages and Operating Systems. ACM, 715–731 (2019)

[134] Christensen, D.V., Dittmann, R., Linares-Barranco, B., Sebastian, A., Gallo, M. L., Redaelli, A., Slesazeck, S., Mikolajick, T., Spiga, S., Menzel, S.: 2021 roadmap on neuromorphic computing and engineering. arXiv preprint arXiv:210505956 (2021)

[135] Upadhyay NK, Jiang H, Wang Z, Asapu S, Xia Q, Joshua YJ (2019). Emerging memory devices for neuromorphic computing. Adv. Mater. Technol..

[136] Sung C, Hwang H, Yoo IK (2018). Perspective: a review on memristive hardware for neuromorphic computation. J. Appl. Phys..

[137] Zhang W, Gao B, Tang J, Yao P, Yu S, Chang MF, Yoo HJ, Qian H, Wu H (2020). Neuro-inspired computing chips. Nat. Electron..

[138] Zhou, Y., Xu, N., Gao, B., Zhuge, F., Tang, Z., Deng, X., Li, Y., He, Y., Miao, X.: Complementary memtransistor-based multilayer neural networks for online supervised learning through (anti-) spike-timing-dependent plasticity. IEEE Trans. Neural Netw. Learn. Syst. (2021) 10.1109/TNNLS.2021.3082911 34081587

[139] Pedretti G, Milo V, Ambrogio S, Carboni R, Bianchi S, Calderoni A, Ramaswamy N, Spinelli AS, Ielmini D (2017). Memristive neural network for on-line learning and tracking with brain-inspired spike timing dependent plasticity. Sci. Rep..

[140] Lu YF, Li Y, Li H, Wan TQ, Huang X, He YH, Miao X (2020). Low-power artificial neurons based on Ag/TiN/HfAlO_*x*_/Pt threshold switching memristor for neuromorphic computing. IEEE Electron Device Lett..

[141] Wan TQ, Lu YF, Yuan JH, Li HY, Li Y, Huang XD, Xue KH, Miao XS (2021). 12.7 mA/cm^2^ on-current density and high uniformity realized in AgGeSe/Al_2_O_3_ selectors. IEEE Electron Device Lett..

[142] Li X, Tang J, Zhang Q, Gao B, Yang JJ, Song S, Wu W, Zhang W, Yao P, Deng N, Deng L, Xie Y, Qian H, Wu H (2020). Power-efficient neural network with artificial dendrites. Nat. Nanotechnol..

[143] He Y, Jiang S, Chen C, Wan C, Shi Y, Wan Q (2021). Electrolyte-gated neuromorphic transistors for brain-like dynamic computing. J. Appl. Phys..

[144] Roy K, Jaiswal A, Panda P (2019). Towards spike-based machine intelligence with neuromorphic computing. Nature.

[145] Chakraborty I, Jaiswal A, Saha A, Gupta S, Roy K (2020). Pathways to efficient neuromorphic computing with non-volatile memory technologies. Appl. Phys. Rev..

